# Hepatocyte dedifferentiation in 2D culture reveals extensive transcriptomic and proteomic rewiring

**DOI:** 10.1097/HC9.0000000000000795

**Published:** 2025-10-07

**Authors:** Morten Dall, Ben Stocks, Daniel T. Cervone, Atul S. Deshmukh, Jonas T. Treebak

**Affiliations:** Novo Nordisk Foundation Center for Basic Metabolic Research, University of Copenhagen, Copenhagen, Denmark

**Keywords:** dedifferentiation, hepatocytes, liver, multi-omics, proteomics, RNAseq, single-nucleus RNAseq

## Abstract

**Background:**

Primary hepatocytes are commonly used in vitro to model liver metabolism, but prolonged culturing results in dedifferentiation and potentially limits the applicability of this model.

**Methods:**

We characterized the transcriptome and proteome of full liver and primary hepatocytes as either freshly isolated cells or after 24 hours of 2D-culturing.

**Results:**

We found that 2D-culturing for 24 hours changes more than 10,000 genes and 3000 proteins compared with freshly isolated cells, accompanied by a decrease in transcriptional heterogeneity and a loss of zonal markers. Moreover, there were changes in proteins associated with the extracellular matrix, in mitochondrial and ribosomal protein abundances, as well as an increase in the abundance of acute-phase response proteins.

**Conclusion:**

Collectively, primary mouse hepatocytes in culture rewire the transcriptome and proteome, which may affect the utility of this model to study physiological and molecular mechanisms related to the liver. We developed the Shiny app “Hepamorphosis” (https://cbmr.ku.dk/research/resources/shiny-apps/), which allows users to explore RNA/protein correlations, zonation profiles, and cell-type-specific transcription in full liver and cultured hepatocytes.

## Graphical abstract

**Figure:**
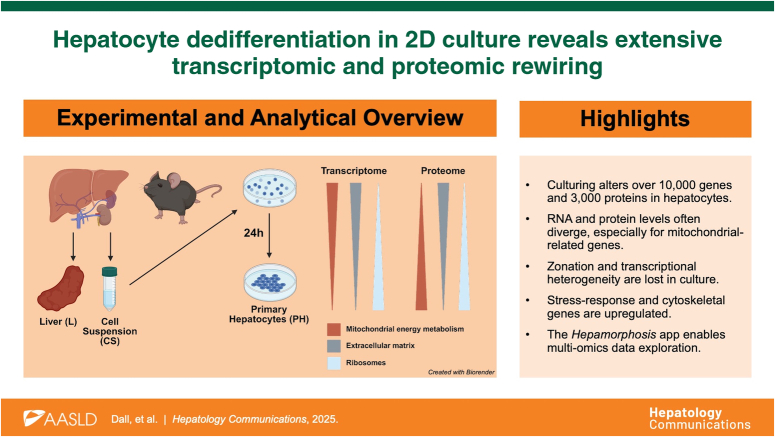

## INTRODUCTION

The liver is central to whole-body glucose and lipid homeostasis, and dysregulation of its metabolic function can lead to metabolic dysfunction–associated fatty liver disease (MAFLD).^1^^,^^2^ Hepatocytes make up over 80% of liver mass in rats^3^ and perform essential roles including plasma protein synthesis, gluconeogenesis, lipid metabolism, bile acid synthesis, and detoxification.^1^ These functions are spatially organized within the liver lobules.^4^ Oxygen-rich blood enters through the periportal area, where hepatocytes predominantly carry out β-oxidation and gluconeogenesis, while pericentral hepatocytes express genes linked to glycolysis, triglyceride synthesis, lipogenesis, and ketogenesis.^4^ Notably, *Cyp2f2* and *Glul* are expressed around the portal and central veins, respectively.^5^ Single-cell RNA sequencing has further delineated 9 lobule layers with zonal gene expression,^5^ a pattern also confirmed at the protein level via single-cell mass spectrometry.^6^

This spatial heterogeneity poses challenges for modeling liver function in vitro. Primary hepatocytes, particularly from human donors, are widely used in research.^7^ In rodents, the preferred method of isolation is 2-step collagenase digestion,^8^ involving sequential perfusion with calcium-free and collagenase buffers, followed by cell seeding on collagen. Rodent hepatocytes are more readily available than human ones and retain key functions like gluconeogenesis and ureagenesis.^9^^,^^10^ However, they undergo dedifferentiation in culture. For instance, one study observed morphological changes in human hepatocytes after just 3 days,^11^ and another reported increased numbers of differentially abundant proteins over time.^12^

In a previous study, we compared the transcriptome of whole liver tissue to that of cultured hepatocytes and observed significant changes, suggesting dedifferentiation during culture.^13^ However, that study had 2 limitations: first, bulk RNA sequencing could not distinguish whether observed transcriptional changes were due to dedifferentiation or altered cell-type composition; second, mRNA and protein levels often show poor correlation,^14^ limiting conclusions about functional consequences.

To overcome these limitations, we expanded our bulk RNAseq analysis and repeated hepatocyte isolation and culturing, this time analyzing samples using both proteomics and single-nucleus RNA sequencing (snRNAseq). This integrated approach allowed us to attribute transcriptional changes to specific cell types and assess how isolation and culture affect the hepatocyte proteome. Our findings reveal that culturing leads to substantial reprogramming of the hepatocyte transcriptome and proteome, including a loss of zonation markers.

## METHODS

### Study approval

All animal experiments were performed in accordance with the European Directive 2010/63/European Union of the European Parliament and the Council for the protection of animals used for scientific purposes. The study was performed according to the ARRIVE guidelines, and ethical approval was given by the Danish Animal Experiments Inspectorate (#2020-15-0201-00764).

### Animal experiments

Male C57BL/6NTac mice were ordered from Taconic at 9 weeks of age. Mice were habituated for a minimum of 5 days before hepatocyte isolation. Mice were group-housed and had ad libitum access to water and chow (D30 Fortified, Safe Diets). Mice were housed in a temperature-controlled environment (22±1C) with a 12-hour light/dark cycle.

### Primary hepatocyte isolation

Hepatocytes were isolated from mice between 10 and 12 weeks of age. Hepatocytes were isolated as previously described,^13^^,^^15^ and the methodological details are provided in the Supplemental Methods description, http://links.lww.com/HC9/C77.

### Bulk RNAseq analysis

RNAseq data were obtained from the Gene Expression Omnibus database (GSE173406) and were originally analyzed in a different study.^13^ Data were filtered to only include data from control animals. Differentially expressed genes underwent gene ontology enrichment analysis using R package ClusterProfiler version 4.10.0,^16^ with the list of mRNAs after expression filtering as background reference. Dotplots display the most significantly enriched targets, automatically selected by the enrichPlot package v. 1.22.0. Significantly enriched GO terms were subsequently clustered using the Leiden algorithm^17^ at resolution 0.6, through the package leidenAlg v. 1.1.5, and cluster names were assigned based on maximal node centrality.

### Proteomics analysis

A detailed description of the proteomics methods is provided in the Supplemental Methods description, http://links.lww.com/HC9/C77.

### Single-nucleus RNA sequencing

A detailed description of the single-nucleus RNA sequencing (snRNAseq) methods is provided in the Supplemental Methods description, http://links.lww.com/HC9/C77.

### Pseudo-bulk analysis

For pseudo-bulk analysis, counts from the Seurat object were saved with metadata as a single-cell object using the SingleCellExperiment package (Version 1.18.0).^18^ Cell-wise quality control and outlier removal were performed using scuttle v. 1.6.3.^19^ Genes with fewer than 10 counts were removed. Counts were obtained using the counts function from the SingleCellExperiment package and were aggregated into a count matrix using the aggregate matrix function from Matrix.utils (v.0.9.8). Counts and metadata were assembled into a DESeqDataSet object and were subsequently analyzed using the DeSeq2 package v1.36.0.^20^ The design matrix was constructed as ~group, where group is sample type (L, CS, or PH).

### Data availability and large language model use

Bulk RNAseq data are available through the Gene Expression Omnibus database (GSE173406). Proteomics data were deposited in the ProteomeXchange Consortium (http://proteomecentral.proteomexchange.org) via the PRIDE partner repository under PRIDE ID (PXD068742). Single-nucleus RNAseq data were uploaded to the Gene Expression Omnibus database (GSE280301). The large language model Microsoft365 Copilot (GPT-4) was used for code debugging, and Open-AI ChatGPT was used to make the language concise.

## RESULTS

### Primary hepatocyte isolation and culturing impact the transcriptome

**FIGURE 1:**
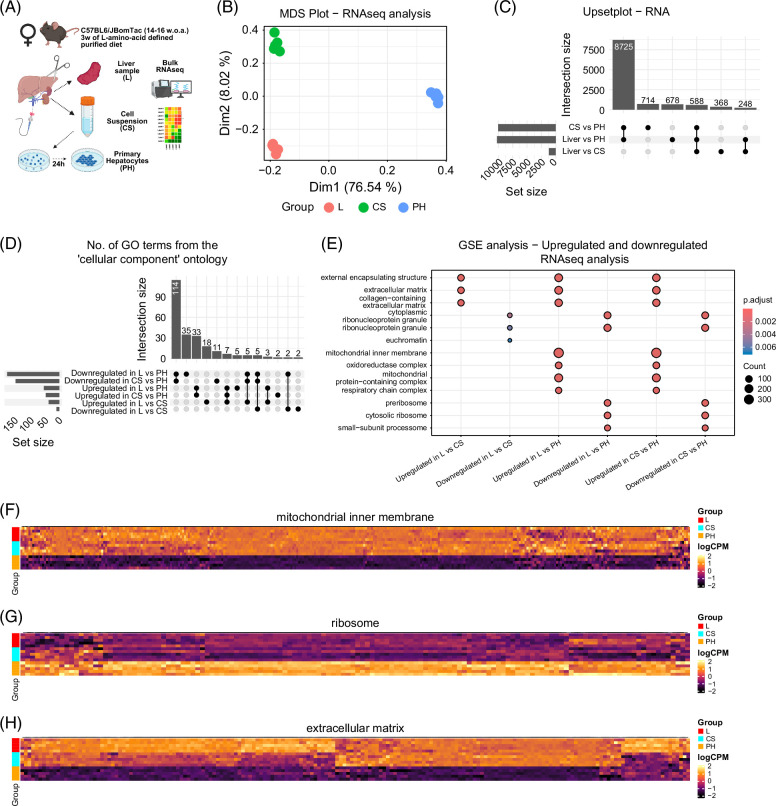
Primary hepatocyte culturing causes major changes to the hepatocyte transcriptome. (A) Overview of the experiment (created in BioRender). (B) MDS plot for dimensions 1 and 2. (C) Upset plot showing overlap of genes with differential expressions for various comparisons. (D) Upset plot showing overlap of significantly enriched gene ontology enrichment terms between comparisons from the *cellular component* ontology. Genes were separated into upregulated or downregulated before gene ontology enrichment analysis. (E) Dotplot showing the top 3 gene ontology enrichment terms from the cellular component ontology. Differentially expressed genes from each comparison were separated into either upregulated or downregulated for each comparison before gene ontology enrichment analysis. (F–H) Heatmaps of differentially expressed genes between Liver and Primary Hepatocytes from the GOCC terms “GO:0005743 - mitochondrial inner membrane,” “GO:0005840 - ribosome,“ and “GO:0031012 - extracellular matrix.” Abbreviations: CS, cell suspension; GOCC, gene ontology cellular component; GSE, gene set enrichment; L, liver; MDS, multidimensional scaling; PH, primary hepatocytes cultured for 24 hours.

To assess how isolation and culturing affect the hepatocyte transcriptome, we re-analyzed a previously published RNAseq dataset^13^ comprising samples from liver biopsies (L), freshly isolated liver cells in suspension (CS), and hepatocytes cultured for 24 hours on collagen (PH) (Figure 1A). All samples were from female C57BL/6JBomTac mice (14–16 weeks of age) fed an L-amino-acid defined low-fat diet for 4 weeks. Multidimensional scaling (MDS) revealed distinct clustering by sample type (Figure 1B). Over 10,000 genes were differentially expressed between Primary Hepatocytes and both Liver and Cell Suspension, with ~8700 genes overlapping, indicating major transcriptional changes due to culturing (Figure 1C and Supplemental File S1, http://links.lww.com/HC9/C78). Around 1000 genes also differed between Cell Suspension and Liver, highlighting the isolation process itself alters gene expression. Volcano plots identified key transcripts contributing to these changes (Supplemental Figure S1A–C, http://links.lww.com/HC9/C79). Hemoglobin genes (*Hbb*, *Hba*) showed reduced expression in Cell Suspension and Primary Hepatocytes compared with Liver, reflecting the removal of blood cells. Primary Hepatocyte samples showed elevated expression of the liver injury marker *Gsta1*,^21^ and reduced expression of endothelial and metabolic genes *Clec4g* and *Cyp7a1*, the latter being crucial in bile acid synthesis.^22^^,^^23^ Gene ontology (GO) enrichment analysis of differentially expressed genes revealed biological processes altered during dedifferentiation (Figure 1D and Supplemental File S2, http://links.lww.com/HC9/C80). Genes downregulated in Primary Hepatocytes were enriched for mitochondrial-associated terms (Figures 1E, F), while upregulated genes were associated with ribosomal components (Figures 1E, G). Genes enriched in Liver samples were linked to extracellular matrix (ECM) organization (Figures 1E, H). To further dissect the data, enriched GO terms were clustered using the Leiden algorithm (Supplemental Figures S1D–I, http://links.lww.com/HC9/C79). In Liver versus Cell Suspension, clusters were associated with ECM and plasma membrane structures. In Liver versus Primary Hepatocytes, most clusters upregulated in Liver related to mitochondrial components and energy metabolism. In contrast, upregulated genes in Primary Hepatocytes were linked to transcription, translation, and cytoskeletal structures. Similar patterns were observed when comparing Cell Suspension and Primary Hepatocytes. Together, these findings show that isolation and particularly 2D culturing cause major transcriptomic shifts in hepatocytes, with mitochondrial downregulation being a prominent feature.

### Primary hepatocyte isolation and culturing impact the proteome

**FIGURE 2:**
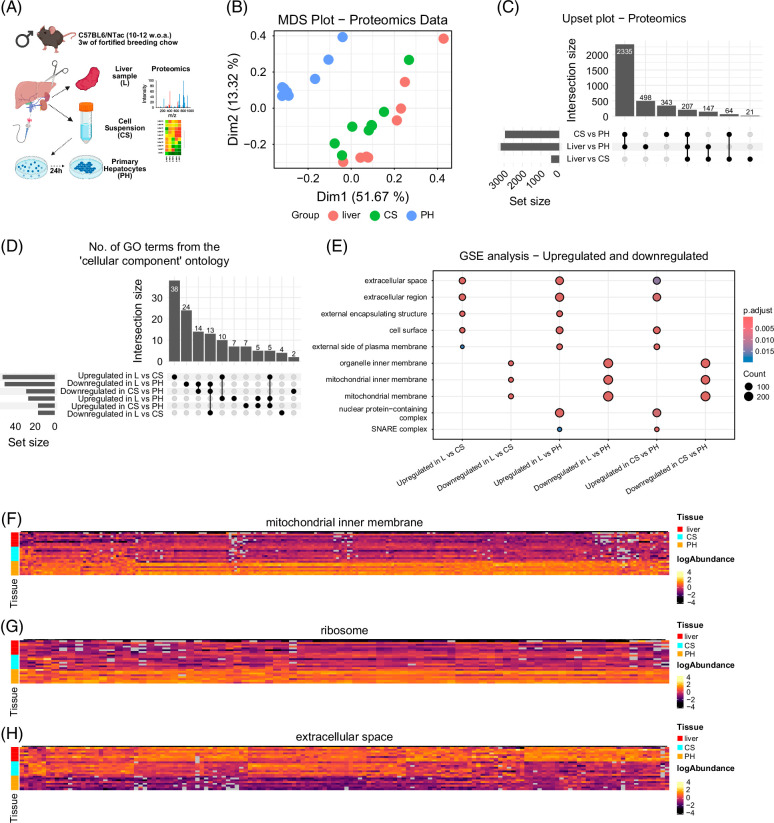
Culturing of primary mouse hepatocytes causes major changes to the hepatocyte proteome. (A) Overview of groups and experimental conditions (created in BioRender). (B) MDS plot for dimensions 1 and 2 showing variation between the 3 groups. (C) Upset plot showing overlap of proteins with differential abundance for various comparisons. (D) Upset plot showing overlap between significantly enriched gene ontology terms from the cellular component ontology. Genes were separated into upregulated or downregulated genes for each comparison before gene ontology enrichment analysis. (E) Dotplot showing the top 5 most significant gene ontology terms from the cellular component ontology for each gene comparison. Genes were separated into upregulated or downregulated genes for each comparison before gene ontology enrichment analysis. (F–H) Heatmaps of differentially expressed genes between Liver and Primary Hepatocytes from the GOCC terms “GO:0005743 - mitochondrial inner membrane,” “GO:0005840 - ribosome,” and “GO:0005615 - Extracellular space.” Abbreviations: CS, cell suspension; GO, gene ontology; GOCC, gene ontology cellular component; GSE, gene set enrichment; L, liver; MDS, multidimensional scaling; PH, primary hepatocytes cultured for 24 hours.

The increased expression of ribosomal genes in cultured hepatocytes suggested altered protein translation and potential shifts in the proteome. To investigate this, we repeated the isolation in a new cohort of chow-fed male C57BL/6NTac mice (10–12 weeks of age), collecting Liver, Cell Suspension, and Primary Hepatocyte samples (Figure 2A). MDS revealed that Primary Hepatocytes separated clearly from both Liver and Cell Suspension, which clustered more closely than in the transcriptomics dataset (Figure 2B). We detected 5755 proteins in Liver, 5676 in Cell Suspension, and 5680 in Primary Hepatocytes. Culturing led to large proteomic changes: ~3000 proteins were differentially abundant between Primary Hepatocytes and both other sample types (Figure 2C and Supplemental File S3, http://links.lww.com/HC9/C81), with 2335 overlapping. Around 400 proteins differed between Liver and Cell Suspension, indicating protein loss during isolation. Collagen subunits were among the most downregulated proteins in Cell Suspension versus Liver, consistent with ECM loss during isolation (Supplemental Figure S2A, http://links.lww.com/HC9/C82). Proteomic differences also reflected cell-type composition: CLEC4F, a KC marker, was reduced in Primary Hepatocytes, while MT1 and MT2—antioxidant proteins induced by cellular stress—were elevated^24^ (Supplemental Figures S2B, C, http://links.lww.com/HC9/C82). We next performed GO enrichment analysis for differentially abundant proteins using the Cellular Component ontology. For Primary Hepatocytes versus Liver, 51 GO terms were enriched in the cultured group (Figure 2D and Supplemental File S4, http://links.lww.com/HC9/C83), many related to ribosomal and mitochondrial components. In contrast, Liver samples showed increased abundance of ECM-related proteins, suggesting breakdown of structural components during isolation (Figure 2E). Interestingly, GO terms associated with mitochondrial membranes were enriched among proteins *decreased* in the Liver relative to both hepatocyte groups (Figures 2E, F), contrasting transcriptomic data showing increased mitochondrial gene expression in liver tissue. This suggests post-transcriptional regulation or altered protein stability. As with RNAseq, ribosomal proteins were more abundant in Primary Hepatocytes, while ECM proteins were reduced (Figures 2G, H). GO term clustering provided further insights (Supplemental Figures S2D–I, http://links.lww.com/HC9/C82): Liver versus Cell Suspension revealed losses in cytoskeletal proteins, while Liver versus Primary Hepatocytes showed enrichment for ECM and adhesion in tissue, and mitochondrial proteins in cultured cells. Collectively, these data confirm substantial remodeling of the hepatocyte proteome during culturing, highlighting discrepancies between transcriptomic and proteomic regulation—particularly regarding mitochondrial components.

### Primary hepatocytes maintain levels of endopeptidases and proteins involved in glutathione transferase activity

Many proteins changed in cultured hepatocytes compared with liver and freshly isolated cells. To assess whether proteins linked to specific cellular functions remained stable post-isolation, we analyzed proteins whose abundance did not change. These were grouped and subjected to gene ontology enrichment analysis. For the *Molecular Function* ontology, 7 GO terms were significantly enriched among unchanged proteins (Supplemental Figure S3A, http://links.lww.com/HC9/C84), primarily related to peptidase activity, especially ubiquitin ligases, suggesting that proteomic changes are mainly due to altered production rather than turnover. Protein abundance from the largest *Catalytic activity, acting on a protein* term, showed no consistent changes in primary hepatocytes, though sample-to-sample variation was observed (Supplemental Figure S3B, http://links.lww.com/HC9/C84). *Glutathione transferase activity* (Supplemental Figure S3C, http://links.lww.com/HC9/C84) and *peptidase activity* (Supplemental Figure S3D, http://links.lww.com/HC9/C84) were also enriched, with some variability. These findings suggest cultured primary hepatocytes maintain peptidase abundance and may be suitable for studying protein degradation.

### Transcriptomics analysis confirms decreased expression of mitochondria-associated genes in isolated hepatocytes

**FIGURE 3:**
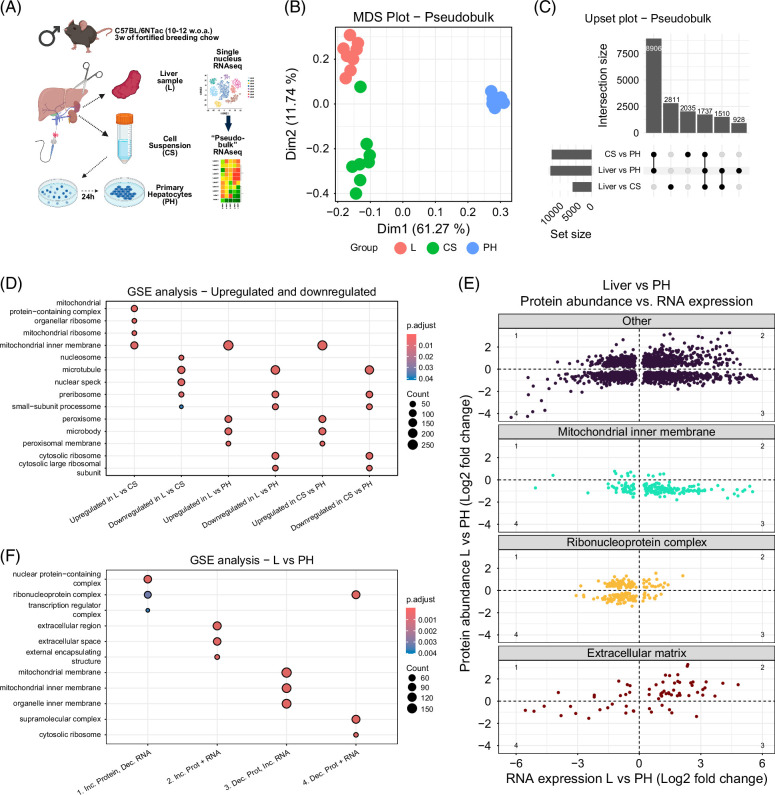
Comparison of proteomics and pseudo-bulk RNAseq data shows a discrepancy between mRNA and protein levels for mitochondrial genes. (A) Overview of experimental groups (created in BioRender). (B) MDS plot for dimensions 1 and 2 for snRNAseq data analyzed as pseudo-bulk. (C) Upset plot showing overlap for differentially expressed genes between various comparisons. (D) Dotplot showing the most significantly enriched gene ontology terms from the cellular component ontology for differentially expressed genes from various comparisons. Differentially expressed genes were separated as either upregulated or downregulated before gene ontology enrichment analysis. (E) Log2-fold change for gene expression in the pseudo-bulk RNAseq dataset plotted against log2-fold change for protein abundance when comparing expression/abundance in liver versus cultured primary hepatocytes. All proteins and genes were differentially expressed and differentially abundant post-FDR correction. Dots from each category were colored and faceted according to relation to specific gene ontology terms, or grouped together as “other.” (F) Dotplot showing the top 3 most significantly enriched gene ontology terms from the cellular component ontology, from genes with a differential expression and differential protein abundance between Liver and Primary Hepatocytes. Genes were separated into 4 groups before analysis: (1) Inc. Prot, Dec. RNA = Log2FCRNA <0 and Log2FRCProtein >0. (2) Inc. Prot + RNA = Log2FCRNA>0 and Log2FRCProtein >0. (3) Dec. Prot, Inc. RNA = Log2FCRNA >0 and Log2FRCProtein <0. (4) Dec. Prot + RNA = Log2FCRNA <0 and Log2FRCProtein <0. Abbreviations: CS, cell suspension; GSE, gene set enrichment; L, liver; MDS, multidimensional scaling; PH, primary hepatocytes cultured for 24 hours; snRNAseq, single-nucleus RNA sequencing.

Transcriptomics and proteomics analyses were conducted on tissues and cells from 2 different mouse models that varied in strain, sex, and diet, which could confound direct comparisons between proteomic and transcriptomic data. To reduce this potential bias, we performed single-nucleus RNA sequencing (snRNAseq) on the same samples used for proteomics (Figure 3A). This approach enabled us to assess transcriptomic overlap with the first cohort and determine whether differences between liver tissue and isolated hepatocytes were due to changes in cell-type composition or adaptation to cell culture. Transcriptomics data were analyzed in pseudo-bulk, where reads from individual cells were aggregated to resemble bulk RNAseq. This analysis confirmed substantial transcriptional changes in hepatocytes following isolation and culturing (Figure 3B), with more than 10,000 differentially expressed genes identified between Primary Hepatocytes and Liver, and between Cell Suspension and Primary Hepatocytes (Figure 3C and Supplemental File S5, http://links.lww.com/HC9/C85). Compared with the first cohort, we observed a greater number of differences between Liver and Cell Suspension, with over 5000 genes affected. Gene ontology enrichment analysis of upregulated and downregulated genes supported previous findings: genes downregulated in the Liver were enriched for ribosomal subunits and microtubules, while upregulated genes were associated with peroxisomes and the *mitochondrial inner membrane* (Figure 3D and Supporting File S6, http://links.lww.com/HC9/C86).

### Genes associated with the mitochondrial inner membrane show increased protein abundance but decreased gene expression in cultured hepatocytes compared with the full liver

Having both snRNAseq and proteomics data from the same samples enabled direct comparison of gene expression and protein abundance for targets detected in both datasets. We visualized log2-fold changes in expression and abundance for genes differentially expressed and abundant between Liver and Primary Hepatocytes. To explore functional trends, we highlighted genes associated with the GO terms *Mitochondrial inner membrane*, *Ribonucleoprotein complex*, and *Extracellular matrix*, with remaining targets plotted separately (Figure 3E). While many genes showed a positive correlation between mRNA and protein changes, a substantial number showed an inverse relationship, with opposing fold-change directions between RNA and protein. Based on these patterns, targets were classified into 4 groups: (1) increased protein abundance but decreased gene expression in Liver; (2) increased gene expression and protein abundance in Liver; (3) increased gene expression but decreased protein abundance in Liver; and (4) decreased gene expression and protein abundance in Liver (Figure 3E). Notably, several *Mitochondrial inner membrane* genes exhibited reduced protein abundance but increased RNA levels in Liver versus Primary Hepatocytes. To further explore this, we performed GO enrichment analysis for *Cellular components* across the 4 groups. Genes with increased transcription and protein abundance in the Liver were enriched for ECM and cell surface terms, while those enriched in Primary Hepatocytes were associated with ribosomal structures (Figure 3F and Supplemental File S7, http://links.lww.com/HC9/C87). Strikingly, genes with increased RNA expression in the Liver but higher protein abundance in Primary Hepatocytes were enriched for mitochondrial terms. These findings suggest that an inverse relationship between transcript and protein levels is a feature of many mitochondrial genes when comparing Liver and cultured Primary Hepatocytes.

### snRNAseq analyses suggest loss of transcriptional heterogeneity in cultured hepatocytes

**FIGURE 4:**
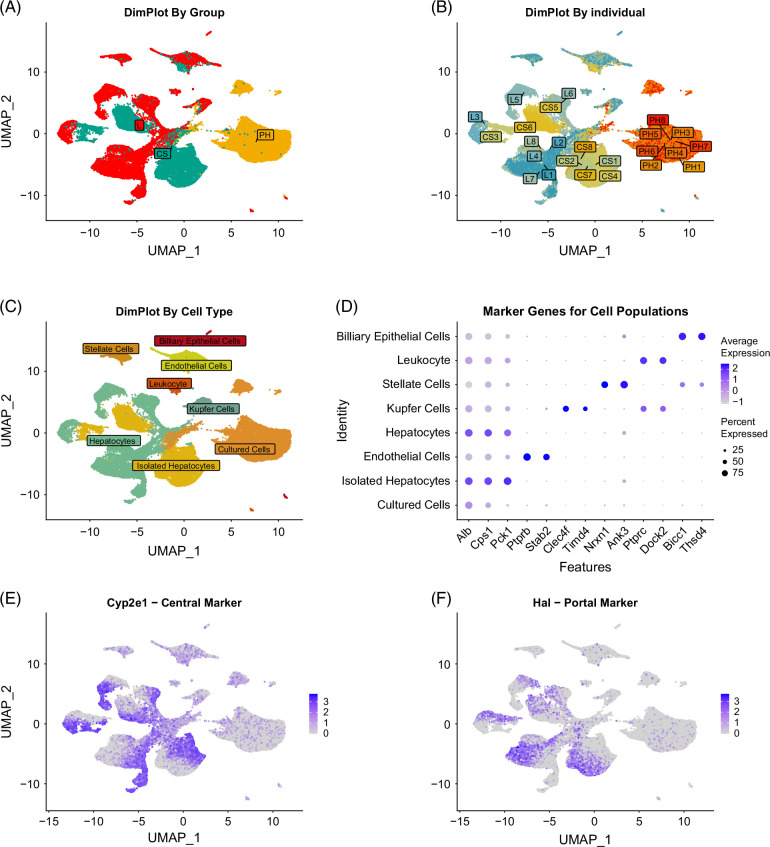
Single-nucleus RNAseq analysis shows decreased heterogeneity and loss of zonation markers in primary mouse hepatocytes following 24 hours of culturing. (A) UMAP projection for dimensions 1 and 2 of snRNAseq data, colored by group. (B) UMAP projection for dimensions 1 and 2 colored by sample. (C) UMAP projection of dimensions 1 and 2 colored by identified cell type. (D) Dotplot showing expression of marker genes used to annotate clusters to specific populations. (E) Feature plot showing expression of the central vein-associated marker gene cytochrome p450 2e1 (Cyp2e1). (F) Feature plot showing expression of the portal-vein-associated marker histidine ammonia lyase (Hal). Abbreviations: CS, cell suspension; L, liver; PH, primary hepatocytes cultured for 24 hours; snRNAseq, single-nucleus RNA sequencing; UMAP, Uniform Manifold Approximation and Projection.

Having confirmed our earlier transcriptomic findings, we next examined how culturing affects cellular diversity in Primary Hepatocyte samples. Uniform Manifold Approximation and Projection (UMAP) visualization of snRNAseq data revealed more distinct clusters in Liver and Cell Suspension samples compared with Primary Hepatocytes, which mainly formed 1 dominant cluster and 2 smaller ones (Figure 4A). This indicates that Primary Hepatocytes exhibit a more homogeneous transcriptional profile. In contrast, clustering by mouse ID was evident in subsets of Liver and Cell Suspension samples (notably from mice 3, 5, and 6; Figure 4B), and became more pronounced when split by sample type (Supplemental Figure S4, http://links.lww.com/HC9/C88). No mouse-specific clustering was observed in Primary Hepatocytes, suggesting that culturing eliminates donor-specific transcriptional variation. Several small clusters in Liver and Cell Suspension were identified as distinct cell types—stellate cells, endothelial cells, leukocytes, KCs, and biliary epithelial cells—based on marker gene expression (Figures 4C, D) and the liver cell atlas (https://www.livercellatlas.org/).^25^ Biliary cells were further confirmed using meta-analysis-derived markers.^26^ Some Primary Hepatocyte cells clustered with hepatocytes from Liver and Cell Suspension, suggesting variable dedifferentiation or potential demultiplexing errors. Cells in Primary Hepatocyte clusters showed reduced expression of hepatic markers such as *Alb*, *Cps1*, and *Pck1* (Figure 4D). Further analysis of hepatocyte clusters from Liver and Cell Suspension showed spatial stratification based on zonation markers like *Cyp2e1* (pericentral) and *Hal* (periportal)^27^ (Figures 4E, F). These zonation markers were low or absent in Primary Hepatocytes, indicating a loss of spatial identity upon culturing. When hepatocytes from all conditions were analyzed together, Primary Hepatocytes showed no clustering by mouse ID or zonation, unlike Liver and Cell Suspension samples (Supplemental Figures 5A–D, http://links.lww.com/HC9/C89). Overall, these results show that spatial position within the liver lobule drives intra-sample clustering, while sample type and donor contribute to inter-sample differences. Culturing induces substantial transcriptional reprogramming, resulting in reduced heterogeneity and loss of spatially defined gene expression profiles in Primary Hepatocytes.

### The transcriptional shift in cultured hepatocytes is associated with an increased expression of ribosomal and cytoskeletal genes

**FIGURE 5:**
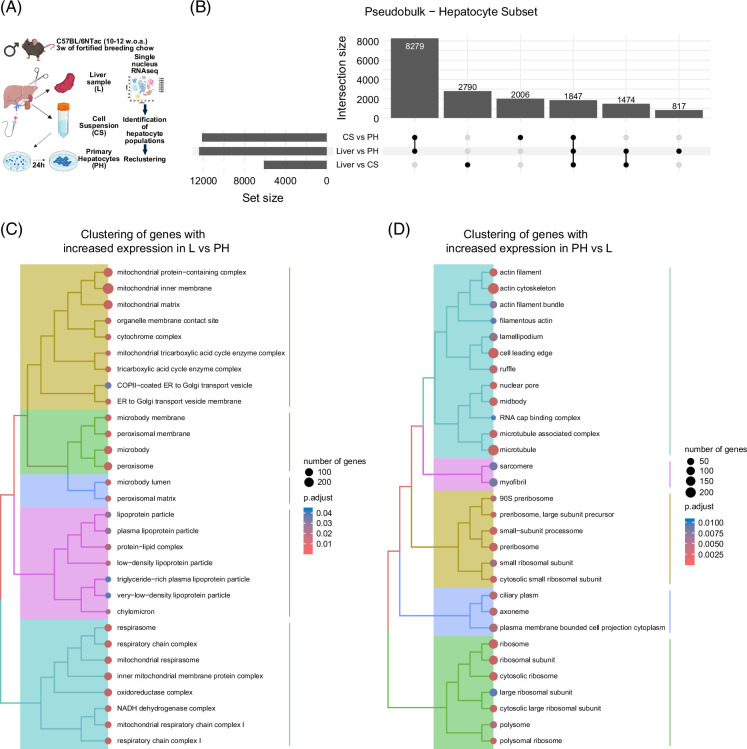
Pseudobulk analysis of hepatocyte gene expression confirms that major changes to the transcriptome following culturing are driven by changes in hepatocyte gene expression. (A) Experimental and analytical overview (created in BioRender). (B) Upset plot showing the number of differentially expressed genes between various comparisons. (C) Treeplots show the clustering of significantly enriched GO terms from the cellular component ontology for genes with increased expression in Liver versus Primary Hepatocytes. (D) Treeplots show the clustering of significantly enriched GO terms from the cellular component ontology for genes with increased expression in Primary Hepatocytes versus Liver. Abbreviations: GO, gene ontology; L, liver; PH, primary hepatocytes cultured for 24 hours.

To assess transcriptional changes in cultured hepatocytes without interference from non-hepatocyte populations, we performed pseudo-bulk analysis on hepatocyte subsets from Liver, Cell Suspension, and Primary Hepatocytes. Over 12,000 genes were differentially expressed between Primary Hepatocytes and both Liver and Cell Suspension samples (Figure 5A and Supplemental File S8, http://links.lww.com/HC9/C90). Genes upregulated and downregulated in Liver versus Primary Hepatocytes were analyzed for enrichment of *Cellular Component* GO terms, followed by clustering. Genes upregulated in the Liver were enriched for mitochondrial energy metabolism, confirming earlier observations (Figure 5B and Supplemental File S9, http://links.lww.com/HC9/C91). In contrast, genes upregulated in Primary Hepatocytes clustered around ribosomal and cytoskeletal components (Figure 5C and Supplemental File S9, http://links.lww.com/HC9/C91), suggesting increased ribosome biogenesis and cytoskeletal remodeling. This aligns with prior findings that dedifferentiation of Primary Hepatocytes involves mechanical tension and stress fiber formation.^28^ Notably, the GO term *Actin cytoskeleton* was enriched in genes upregulated in Primary Hepatocytes compared with Liver (Figure 5C). These results suggest that cultured hepatocytes increase expression of cytoskeletal genes to adapt to their *in vitro* environment. Overall, the pseudo-bulk analysis confirms that key transcriptional changes in ribosomal and cytoskeletal genes occur within hepatocytes during adaptation to culture.

### Primary hepatocyte cultures contain dedifferentiated endothelial cells

**FIGURE 6:**
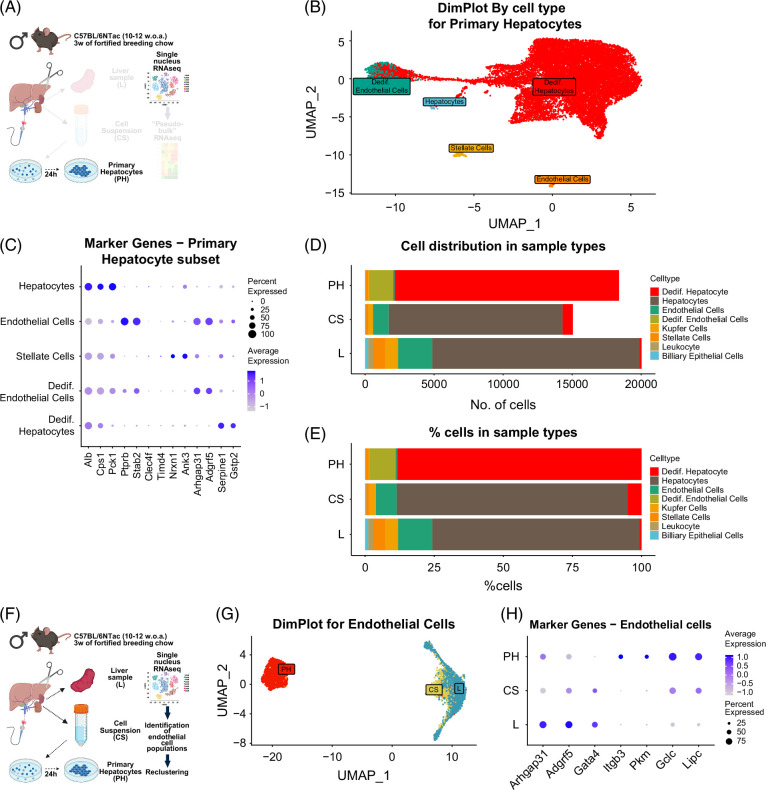
Primary hepatocyte culture contains a subpopulation of dedifferentiated endothelial cells in addition to dedifferentiated hepatocytes. (A) Schematic overview of cultured hepatocyte subsetting and analysis (created in BioRender). (B) UMAP plot showing clustering of cells from Primary Hepatocytes samples. Identities were assigned to identified Seurat clusters, based on expression of known marker genes. (C) Dotplot showing marker genes, used to assign identities to Seurat clusters. (D) Bar graph showing the number of cells annotated to various cell types through marker gene expression. (E) Cellular composition of sample types is displayed as a percentage. (F) Schematic overview of endothelial cell identification and reclustering (created in BioRender). (G) UMAP plot showing clustering of subset endothelial cells from Cell Suspension, Liver, and Primary Hepatocytes. (H) Marker genes for normal and dedifferentiated endothelial cells in the 3 groups. Abbreviations: L, liver; CS, cell suspension; PH, primary hepatocytes cultured for 24 hours; UMAP, Uniform Manifold Approximation and Projection.

Cells from Primary Hepatocytes primarily clustered into one major and two smaller groups (Figure 4A). To determine if all clusters were hepatocyte-associated, we re-clustered cells from Primary Hepatocyte samples (Figure 6A), confirming one dominant population and several smaller ones (Figure 6B). Marker gene expression analysis (Figure 6C) revealed three main cell types: dedifferentiated hepatocytes, dedifferentiated endothelial cells, and stellate cells. Some smaller clusters resembled non-cultured hepatocytes and endothelial cells, potentially representing either partially differentiated cells or misassigned cells from Liver and Cell Suspension samples. Dedifferentiated endothelial cells were identified by expression of markers such as *Arhgap31* and *Adgrf5*. Thus, Primary Hepatocyte cultures contain a small population of endothelial cells that undergo similar dedifferentiation. Leukocytes, KCs, and biliary epithelial cells were absent, and stellate cells were significantly reduced (Figures 6D, E). Hepatocytes made up 75% of cells in the Liver, but 90% in both Primary Hepatocytes and Cell Suspension, indicating enrichment through isolation. Endothelial cell proportions remained comparable across samples (12.3% in Liver vs. 9.4% in Primary Hepatocytes), confirming that cultured hepatocyte samples retain a notable fraction of endothelial cells.

### Dedifferentiated endothelial cells undergo transcriptional changes to adapt to culturing conditions

To investigate endothelial dedifferentiation in culture, we clustered endothelial cells from Liver, Cell Suspension, and the smaller Primary Hepatocyte cluster identified as endothelial (Figure 6F). Dedifferentiated endothelial cells from Primary Hepatocytes were clearly separated from those in Liver and Cell Suspension (Figure 6G). While endothelial markers such as *Arhgap31* and *Adgrf5* were still expressed, their levels were markedly reduced (Figure 6H). The liver-specific endothelial marker *Gata4* was only expressed in Liver and Cell Suspension, further supporting dedifferentiation in cultured cells (Figure 6H).^29^ Primary Hepatocyte-derived endothelial cells expressed high levels of integrin beta-3 (*Itgb3*), likely reflecting adaptation to collagen-coated culture surfaces, and pyruvate kinase (*Pkm*), indicating a shift toward glycolytic metabolism. They also expressed high levels of *Gclc*, the rate-limiting enzyme in glutathione biosynthesis, suggesting enhanced oxidative stress defenses. Notably, they expressed *Lipc*, a triacylglycerol lipase typically found in hepatocytes and endothelial cells^30^ (Figure 6H). These findings demonstrate that cultured Primary Hepatocytes include endothelial cells that undergo transcriptional dedifferentiation in response to 2D culture conditions.

### Culturing of primary hepatocytes induces altered expression of glucose transporters and increased abundance of proteins associated with oxidative stress

**FIGURE 7:**
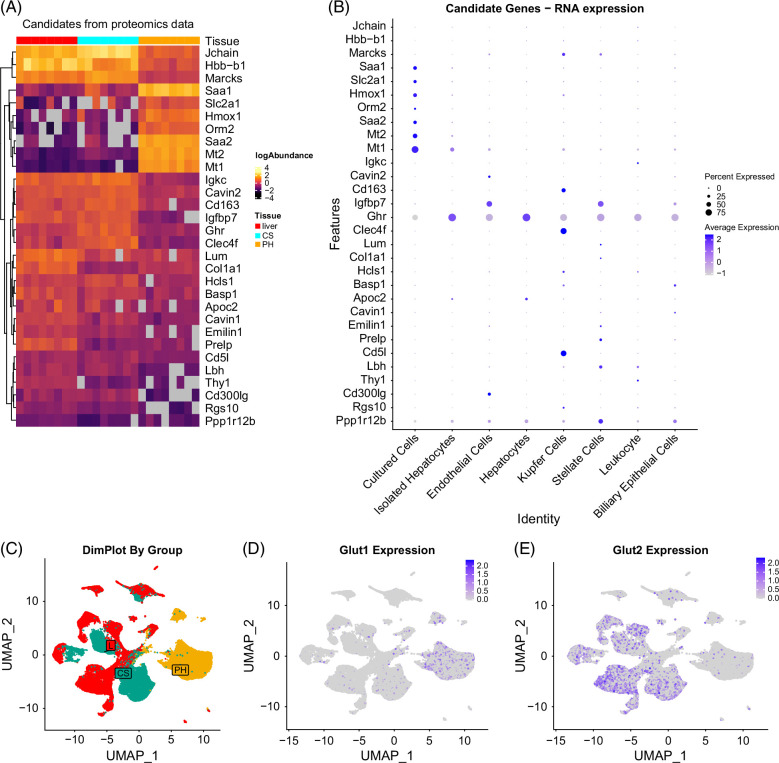
Analysis of the most highly abundant proteins in Primary Hepatocytes suggests oxidative stress. (A) Heatmap showing protein abundance of candidate proteins with a log FC >2 or log FC <−2 for both RNA levels and protein abundance for Liver vs. Primary Hepatocytes. (B) Dotplot showing cell-type-specific expression levels of candidate genes. (C) UMAP plot colored by sample type. (D) UMAP plot of all samples colored by expression of *Glut1*. (E) UMAP plot of all samples colored by expression of *Glut2*. Abbreviation: UMAP, Uniform Manifold Approximation and Projection.

The combination of snRNAseq and proteomics enabled us to annotate high-abundance proteins to specific cell types. We selected candidate proteins based on three criteria: (1) significantly altered abundance in Primary Hepatocytes versus Liver, (2) concordant mRNA and protein fold changes (ie, both increased or both decreased), and (3) a log2-fold change >2 or <−2. This yielded 30 candidate proteins (Figure 7A). Among these, COL1A1, LUM, and PRELP—ECM proteins—were decreased in Primary Hepatocytes, consistent with loss during cell isolation.^31^^,^^32^ Proteins reduced in cultured hepatocytes also included markers of specific non-parenchymal cells: *Igfbp7* (endothelial and stellate), *Cd163*, *Clec4f*, and *Cd5l* (Kupffer) (Figure 7B), suggesting that their lower abundance reflects reduced representation of these cell types in culture. Conversely, seven proteins were more abundant in Primary Hepatocytes, including acute-phase proteins SAA1 and SAA2, and antioxidants MT1 and MT2, consistent with stress responses.^24^^,^^33^ ORM2, another acute-phase protein,^34^ and HMOX-1, an oxidative stress-induced enzyme,^35^ were also elevated, supporting the notion that cultured hepatocytes are under oxidative stress. Notably, *Slc2a1* (*Glut1*) was highly expressed in cultured hepatocytes (Figure 7D), whereas *Slc2a2* (*Glut2*) was predominant in the whole liver (Figure 7E). While both transporters are insulin-independent, GLUT1 has a higher glucose affinity than GLUT2 (1–2 mM vs. 15–20 mM).^36^ This shift in transporter expression may reflect metabolic adaptation to culture, resembling changes seen in cancer cells.^37^ UMAP analysis confirmed that *G* *lut1* expression localized almost exclusively to cultured hepatocytes (Figure 7C). Together, these findings show that hepatocyte culturing leads to loss of cellular diversity, significant changes in protein expression, and increased expression of stress-related and metabolism-related genes.

### Multi-omics data are available for exploration through the “Hepamorphosis” app

To facilitate data exploration, we developed an interactive dashboard—Hepamorphosis—to support hypothesis generation, experimental planning, and quality control. The app includes 3 modules: *Gene Explorer*, *Correlator*, and *Zonator*. *Gene Explorer* enables users to visualize gene expression across cell types using snRNAseq data and, when available, compare corresponding protein abundance across sample types via violin plots (Supplemental Figure S6A, http://links.lww.com/HC9/C92). A UMAP colored by cell type is included for reference, allowing users to assess gene specificity and dedifferentiation effects. *Correlator* links transcriptomic and proteomic data, plotting log fold changes for genes found in both datasets across 3 comparisons: Liver versus Cell Suspension, Liver versus Primary Hepatocytes, and Cell Suspension versus Primary Hepatocytes (Supplemental Figure S6B, http://links.lww.com/HC9/C92). This helps determine whether RNA and protein levels track similarly during dedifferentiation. *Zonator* focuses on simplified hepatocyte zonation. snRNAseq data from Liver hepatocytes were clustered using Seurat and annotated as central or periportal based on zonation markers such as *Cyp2e1* and *Hal*. Zonator displays UMAP and violin plots to compare gene expression across zones (Supplemental Figure S6C, http://links.lww.com/HC9/C92). Together, Hepamorphosis provides a powerful, user-friendly resource for interrogating the effects of hepatocyte culture at both RNA and protein levels.

## DISCUSSION

We set out to investigate how primary mouse hepatocytes dedifferentiate during culture and how cellular heterogeneity is affected by isolation and culturing. We demonstrate that within 24 hours of 2D culture, primary hepatocytes undergo extensive transcriptomic and proteomic changes. This rapid transformation questions whether cultured hepatocytes retain the functional and molecular characteristics of their *in vivo* counterparts. Moreover, zonation-specific markers are largely lost, and cultured hepatocytes coexist with non-hepatocyte populations, further complicating their use to study liver function.

We were aware that gene expression changes rapidly post-isolation, with one study documenting over 1,000 altered transcripts in cultured rat hepatocytes after 24 hours.^38^ These changes are congruent with known morphological shifts, as hepatocytes adopt a flattened, fibroblast-like appearance over time in culture.^39^ However, based on earlier proteomics data from primary human hepatocytes, we expected relative stability in protein abundance. That study found mitochondrial protein levels to be maintained and reported only about 40 proteins as differentially abundant within 24 hours,^12^ suggesting that the proteome was more stable than the transcriptome.

In contrast, our study revealed more extensive remodeling: over 10,000 differentially expressed genes and 3,000 altered proteins after 24 hours of culturing. This suggests that primary mouse hepatocytes dedifferentiate to a greater extent than previously appreciated. While biological differences between mouse and human hepatocytes could contribute to these discrepancies, methodological factors may also play a role. For instance, the human hepatocytes were derived from surgically resected perfused biopsies,^12^ which may be more robust due to the physiological condition of the tissue and the isolation approach. Our method used a standard 2-step collagenase perfusion technique,^8^ a widely adopted protocol for rodent hepatocytes. It remains difficult to directly compare these models, but our results underscore the need for careful interpretation when extrapolating from cultured cells.

A key observation was the consistent downregulation of genes associated with ECM components, beginning even in the cell suspension stage. This likely reflects the loss of HSCs, the principal ECM-producing cell type in the liver.^40^ Dotplots of ECM ontology genes upregulated in Liver versus Cell Suspension samples confirmed that most were specifically expressed in stellate cells (Supplemental Figure S7, http://links.lww.com/HC9/C93). snRNAseq further showed a dramatic reduction in stellate cell representation from Liver to Cell Suspension and an even greater loss in Primary Hepatocytes. These data indicate that much of the observed decrease in ECM gene expression results from cell type loss rather than changes within hepatocytes themselves.

In contrast, we observed a robust increase in ribosomal gene expression in cultured hepatocytes. This likely reflects increased ribosome biogenesis to support the global transcriptional changes occurring during dedifferentiation. This phenomenon appears to be hepatocyte-intrinsic, as it was not observed in Cell Suspension versus Liver samples, which showed minimal ribosomal gene enrichment. Notably, ribosomal protein synthesis, rather than rRNA transcription, is known to be the rate-limiting step in ribosome biogenesis,^41^ consistent with our findings. Proteomics data confirmed these transcriptomic patterns, showing increased ribosomal protein levels and reduced ECM-related protein abundance.

An intriguing observation was the increased abundance of mitochondrial proteins in cultured hepatocytes, contrasting with transcriptomic data and earlier reports that suggested a decline in mitochondrial content during culture.^12^ Thus, we performed snRNAseq on the same cohort used for proteomics, which allowed a more direct comparison between transcript and protein levels. We found that the correlation between RNA expression and protein abundance varied by subcellular localization. For several mitochondrial genes, we observed a negative correlation, suggesting either post-transcriptional regulation or asynchronous changes in mRNA and protein levels. One interpretation is that mitochondrial biogenesis may be initiated early during adaptation to culture, with a lag between mRNA induction and protein accumulation. Once sufficient mitochondrial capacity is reached, mRNA levels may drop while protein levels remain elevated. Time-resolved transcriptomics and proteomics experiments could be used to test this hypothesis.

An increase in mitochondrial proteins raises the question of whether cultured hepatocytes have enhanced respiratory capacity. In a previous study, we assessed oxygen consumption rates (OCR) in cultured hepatocytes using the Seahorse system and in liver biopsies using the Oroboros Oxygraph.^15^ Cultured hepatocytes exhibited a minor decrease in OCR upon ATP synthase inhibition, suggesting partial reliance on glycolysis. However, FCCP-induced uncoupling led to a substantial OCR increase, indicating preserved or enhanced mitochondrial respiratory potential. While informative, direct comparisons between adherent and suspended cells remain challenging due to differing measurement conditions and normalization strategies. Newer adaptations of the Seahorse technology to measure OCR in suspended cells^42^ may allow more direct comparisons, provided normalization issues can be adequately addressed.

snRNAseq data also revealed a substantial reduction in hepatocyte heterogeneity during culture. Cells from Liver and Cell Suspension samples retained zonation-specific expression patterns, consistent with previous findings showing spatial compartmentalization of hepatocyte function. In contrast, Primary Hepatocytes lacked clear zonation signatures. While protocols exist to enrich for pericentral or periportal hepatocytes via digitonin and collagenase perfusion,^43^ our findings suggest that zonation markers are rapidly lost upon culturing. Recent single-cell proteomics studies confirm that roughly half of hepatocyte proteins are zonated along the central-to-portal axis,^6^ implying that zonation loss at the RNA level is likely mirrored at the protein level. This homogenization limits the applicability of 2D cultures for studying spatially restricted liver functions.

Although a recent study suggested that human hepatocytes cultured *in vitro* segregate into distinct subpopulations,^44^ we did not observe such clustering in mouse Primary Hepatocytes. However, we did detect several distinct non-hepatocyte populations. These included endothelial cells, identified by expression of markers like *Ptprb* and *Stab2*, though the expression of these markers was diminished, consistent with dedifferentiation. Stellate cells also remained detectable in culture, albeit in reduced numbers. Altogether, non-hepatocyte populations comprised roughly 10% of cultured cells. Thus, hepatocyte cultures are not entirely pure and coexisting non-parenchymal cells may influence experimental outcomes, particularly in co-culture or conditioned medium experiments.

We also identified proteins that were highly expressed in liver tissue but nearly absent in cultured cells. These included CLEC4F, CD163, and CD5L, markers of KCs, as well as *Ppp1r12b*, *Lbh*, and *Igfbp7*, associated with stellate cells. The absence of these proteins in cultured hepatocytes underscores the importance of accounting for cell-type diversity in liver tissue. By integrating proteomic and snRNAseq data, we were able to attribute protein losses to specific missing cell types rather than to dedifferentiation of hepatocytes themselves. This approach can help distinguish genuine dedifferentiation from artifacts of cell population shifts.

Stress responses were also evident in cultured hepatocytes. Enrichment of actin cytoskeleton–related GO terms and upregulation of acute-phase proteins and metallothioneins suggest that cells experience mechanical and oxidative stress. This is consistent with previous work showing that mechanical tension in 2D cultures drives dedifferentiation, which can be mitigated by mechanical relaxation.^28^ Exposure to atmospheric oxygen levels, substantially higher than physiological liver conditions, may further induce oxidative stress and dedifferentiation.^45^ Culturing in lower oxygen concentrations (e.g., 5%) was previously shown to help preserve hepatocyte identity. Several of the stress–response genes we identified could be developed as biomarkers for hepatocyte health or differentiation state.

Interestingly, not all functions were disrupted by culture. Peptidase activity appeared largely unaffected, suggesting that proteolysis and protein turnover may remain functional in dedifferentiated hepatocytes. This is promising for studies focused on liver proteostasis, drug metabolism, or protease-targeted interventions.

Despite widespread gene and protein changes, pinpointing specific factors driving dedifferentiation remains challenging. Prior studies indicate that dedifferentiation mirrors a reversion to a fetal-like gene expression profile, with fetal markers increasing within 4 hours of culture.^46^ This suggests that strategies to preserve differentiation must be applied immediately upon plating. Our dataset offers a resource for identifying candidate genes and proteins whose modulation could preserve hepatocyte identity. GO analysis highlighted the term *nuclear protein-containing complex* among genes upregulated in culture, suggesting transcriptional regulators that may serve as promising targets in future screens.

While traditional 2D cultures remain standard, 3D models of hepatocyte culture have gained traction in recent years.^47^ These models better preserve *in vivo* gene expression patterns and metabolic functions and are increasingly used to study drug metabolism and toxicity.^48^ However, they still fall short in replicating hepatic zonation,^49^ and their molecular profiles remain incompletely characterized. Future work comparing 2D and 3D systems at the transcriptome and proteome levels will be essential to determine their relative suitability for various applications.

In conclusion, primary hepatocytes undergo profound transcriptional and proteomic remodeling during isolation and 2D culture. This process erases zonal identities, reduces cell-type diversity, and activates stress–response pathways. Nevertheless, some functions remain intact, and our integrated transcriptomic and proteomic resource, including the Hepamorphosis app, provides a valuable tool for evaluating whether specific pathways or processes are preserved *in vitro*. These data will help in assessing the suitability of hepatocyte cultures for specific experimental needs.

## Supplementary Material
