# Supplementary material for: Hepatocyte dedifferentiation in 2D culture reveals extensive transcriptomic and proteomic rewiring
Source: Hepatol Commun. 2025 Oct 7;9(11):e0795. doi: 10.1097/HC9.0000000000000795 (PMC12506984; doi:10.1097/HC9.0000000000000795)
Supplement: Supplementary file 1 [file hc9-9-e0795-s001.docx]

**Hepatocyte dedifferentiation in 2D culture reveals extensive transcriptomic and proteomic rewiring**

**Morten Dall^1^, Ben Stocks^1^, Daniel T. Cervone^1^, Atul S. Deshmukh^1^, Jonas T. Treebak^1*^**

^1.^ Novo Nordisk Foundation Center for Basic Metabolic Research, University of Copenhagen

* Corresponding author:

Jonas T. Treebak

University of Copenhagen

Faculty of Health and Medical Sciences

Novo Nordisk Foundation Center for Basic Metabolic Research

Blegdamsvej 3B

Maersk Tower, 7.7.46

DK2200 Copenhagen

Denmark

[jttreebak@sund.ku.dk](mailto:jttreebak@sund.ku.dk)

## Supplementary Methods descriptions

### Primary hepatocyte isolation

Hepatocytes were isolated from mice between 10 and 12 weeks of age. Hepatocytes were isolated as previously described ^1,2^. Mice were sedated using avertin, prepared from a stock of 1 g/mL tribromoethanol in T-amyl alcohol, diluted 1:20 in saline, and dosed as 10 μL/g body weight. A 24-gauge catheter was inserted into the vena cava, and the liver was perfused with 50 mL Hanks buffered saline solution without calcium and magnesium (HBSS, 14170112, Thermo Fisher Scientific, Waltham, MA) supplemented with 76 mg/L EGTA and 10 mM HEPES. Buffer was kept at 42 °C to account for heat loss, and the liver was perfused at 3.5 mL/min. Once blood was cleared from the liver, a suture was tightened around the median lobe, and a tissue sample of the lobe was cut off and snap-frozen in liquid nitrogen. The liver was subsequently perfused with 50 mL Williams E Medium (32551020, Thermo Fisher Scientific) supplemented with 400 mg/L Collagenase (C5138, Sigma). Hepatocytes were dispensed in a Petri dish with plating medium (Minimum essential medium Eagle-199 (MEM-199) (41150, Thermo Fisher Scientific), 10% FBS, 1% P/S), and the suspension was passed through a 100 µm strainer (352360, Corning, Corning, NY). Cells were centrifuged three times at 50 *g* for 2 min, and between each step the pellet was washed with plating medium. Following the final wash, suspension was passed through a 70 µm strainer (352350, Corning), and viability was assessed (NucleoCounter NC-200, ChemoMetec, Denmark). Cell Suspension samples were prepared by centrifuging 2 mL cell suspension at 100 *g* for 2 min. The supernatant was removed, and the pellet was snap-frozen in liquid nitrogen. Primary hepatocyte samples were prepared by seeding 1 million living cells pr. well in a collagen-coated 6-well plate in plating medium. Cells were allowed a minimum of 2 hours to attach, and attachment was confirmed visually through a microscope. The medium was replaced with Culture Medium (MEM-199 with 0.5% FBS, 1% P/S, 1 µM dexamethasone and 1 nM insulin), and cells were incubated overnight at 37 °C with 5% CO_2_. On the next day, the media was aspirated, and cells were washed with PBS and 200 µL of trypsin-EDTA were added. Cells were incubated for 5 min at 37 °C, and 800 µL culture media was added to each well to neutralize the trypsin. Cells were transferred to a 1.5 mL eppendorph tube, and centrifuged at 200 *g* for 5 min. The supernatant was removed, and pellets were snap-frozen in liquid nitrogen. All samples were stored at -80 °C until further analysis.

### Proteomics analysis

#### Sample preparation for proteome analysis

Samples were homogenized in 4% SDS buffer (0.1 M TrisHCl and 4% SDS), boiled at 95 °C for 10 min and debris was removed by centrifugation (16,000 *g* for 10 min). Lysates were sonicated prior to reduction and alkylation (by addition of 50 mM tris(2-carboxyethyl)phosphine (TCEP) and 55 mM chloroacetamide (CAA)) and processed via acetone-precipitation (protein pellets were resuspended in 1% sodium deoxychlorate (SDC)) and in-solution digestion using LysC and trypsin (1:500 and 1:100 enzyme to protein, respectively). The following morning, samples were acidified with isopropanol containing 10% trifluoroacetic acid (TFA) to halt enzymatic digestion. Peptides were centrifuged for 10 min at 14,000 *g* and then loaded onto (3x) SDB-RPS StageTips for desalting ^3^. Peptides were first cleaned twice with 100 µL isopropanol containing 1% TFA and then subsequently twice with 100 µL isopropanol containing 0.2% TFA. Peptides were eluted in a buffer containing 80% acetonitrile and 1% ammonia. The organic solvents were evaporated in a sample concentrator for 1h at 45 °C, and peptides were then re-suspended in 5% acetonitrile containing 0.1% TFA. 200 ng peptides were loaded onto Evotips (EV2001, Evosep) in accordance with the manufacturer’s instructions.

#### High-pH reversed-phase fractionation for library generation

Samples were pooled within each experimental model (i.e., liver, cell suspension, and primary hepatocytes) for the purpose of generating a comprehensive master spectral library of precursor and fragment ions. For library generation, 20 µg of sample type-specific digested peptides were fractionated using High pH Reverse-Phase Chromatography (HpH-RP) on a Kinetex 2.6 µm EVO C18 100Å, 150 x 0.3 mm column (Phenomenex) using an EASY-nLC 1200 System (Thermo) operating at 1.5 µL/min. A 62 min step-gradient from 3 to 60 % solvent B (10 mM TEAB in 80 % acetonitrile) and solvent A (10 mM TEAB in water) was used for separation. Total run time was 98 min including wash and column equilibration. Eluting peptides were collected each 60 sec, obtaining 98 fractions subsequently concatenated into 24 pooled fractions: i.e., the mobile phases of fractions 1, 25, 49, 73 and 97 were pooled into pooled fraction 1, while fractions 2, 26, 50, 74 and 98 were pooled into pooled fraction 2, and so on until pooled fraction 24. 200 ng of HpH-RP fractionated peptides were loaded, concentrated, and desalted on Evotips following the manufacturer’s instructions.

#### LC-MS/MS Analysis

Peptides were separated on 15 cm, 150 μM ID columns packed with C18 beads (1.5 μm; PepSep) on an Evosep ONE HPLC using the “30 samples per day” method ^4^. Peptides were injected via a CaptiveSpray source and 10 μm emitter into a timsTOF pro2 mass spectrometer (Bruker) operated in DDA-PASEF or DIA-PASEF mode as previously described ^5,6^. Briefly, the DDA-PASEF scan range for both MS and MS/MS was set to 100 - 1700 m/z, and TIMS mobility range to 0.6 – 1.6 (V cm^−2^). TIMS ramp and accumulation times were set to 100 ms each, and 10 PASEF ramps recorded for a total cycle time of 1.17 sec. MS/MS target intensity and intensity threshold were set to 20,000 and 1,000, respectively. An exclusion list of 0.4 min for precursors within 0.015 m/z and 0.015 V cm^−2^ width was also activated. For DIA-PASEF the scan range was set to 400-1,200 (m/z), the TIMS mobility range to 0.6-1.43 (V cm^−2^), and ramp and accumulation times to 100 ms. 32 windows of 25 Da width each were placed in the in the m/z–ion mobility plane. The diaPASEF scheme was set to 2 repetitions of 16-scan. The resulting estimated cycle time was 1.80 sec.

#### Spectral library generation in FragPipe

The FragPipe (v18.0) computational platform nested within MSFragger ^7^ was used to build a concatenated master spectral library from all fractionated samples (i.e., liver, cell suspension, and primary hepatocytes). This was done to not introduce artificial differences/biases during quantification, which would arise had each sample-type been quantified separately. Using raw (.d) files, tandem mass spectra (MS/MS) were used for peptide identification in the MSFragger search engine. Only Swiss-Prot reviewed sequences from the UniProt sequence database for *Mus musculus* (downloaded August 9, 2021, 17,082 entries) were used, as well as a list of common contaminants. Decoy reversed protein sequences were also added to the original databases. For MSFragger analysis, fragment mass tolerances were set to 20 ppm, with spectrum deisotoping, mass calibration, and parameter optimization enabled ^7^. Enzyme specificity was set to “strict trypsin“, and up to two missed cleavages were permissible. Isotope error was set to “0/1/2“, and peptide length was set from 7 to 50. Peptide mass was set from 500 to 5,000 Da. Methionine oxidation and acetylation of N-termini were set as variable modifications. Carbamidomethylation of cysteine residues was set as a fixed modification. The maximum number of variable modifications per peptide was set to 3 and the FDR was controlled at < 1% at the peptide spectrum match and protein levels.

#### Computational LCMS/MS Data Analysis

The FragPipe-generated master spectral library was used in concert with DIA-PASEF mass spectra for analysis in DIA-NN (v1.8). DIA-NN operated in double-pass mode with maximum mass accuracy and MS^1^ tolerances of 10 ppm ^7^. Match-between-runs (MBR) was enabled, similar to previous workflows ^7^. DIA-NN protein inference was from FASTA protein names, and briefly, a hybrid approach was used, which combines features from both the master spectral library and *in silico* digested FASTA sequences coupled to a deep learning-based neural network for peptide identification ^7^. The quantification mode was set to “Robust LC (high precision)”. Enzyme specificity was set to trypsin, defined as C-terminal to arginine and lysine (excluding proline). A maximum of 1 missed cleavage was permitted and a minimum peptide length of seven amino acids was required. Carbamidomethyl cysteine was set as a fixed modification, while N-terminal acetylation and methionine oxidation were set as variable modifications. The false discovery rate (FDR) for precursor identification was set to 1%. All other settings were left as default.

#### Proteomics data analysis

Libraries from the three sample types (liver, cell suspension, and primary hepatocytes) were combined and used to quantify the samples, using the DIA-NN ^7^. The raw label-free intensity matrix was imported into Rstudio, proteins were filtered for a minimum of 50% valid values, and analyzed for differential protein abundance using the R-package Limma version 3.52.2 ^8^. Differentially abundant proteins were subject to gene ontology enrichment analysis using the R package ClusterProfiler version 4.10.0 ^9^, and using the list of proteins detected in the combined library as background reference. Significantly enriched GO-terms were clustered as described for bulk RNAseq.

### Single nucleus RNA sequencing

#### Nuclei isolation

Nucleus suspensions were extracted as previously described ^1^. Following isolation, the nucleus pellets were suspended in 110 µL nucleus buffer (PBS; w/o MgCl₂ and CaCl₂ [20012068; Invitrogen]), 1 % BSA (SRE0036-25 ml; Sigma), 2 mM MgCl_2_ (M1028-100 mL; Sigma), and 0.1 % Protector RNase inhibitor 40 U/μL (3335399001; Sigma), and the solution was passed through a 40 μm strainer (43-10040-40; pluriSelect life Science, Germany). For each sample, 10 µL was recovered and mixed with 10 µL trypan blue solution (93595-50 mL, Sigma) for counting nuclei in a counting chamber. The remaining 100 µL were added 1 µL of individual TotalSeq-A antibodies (BioLegend, San Diego, CA), for a final concentration of 5 ng/µL to enable multiplexing ^10^. Samples were washed, centrifuged, re-suspended, and stored as previously described ^1^.

#### Sequencing

Using a SH800S Cell Sorter (SONY), 6,000 nuclei from 22 of the samples were sorted. For two of the liver samples, only 3,005 and 4,015 nuclei could be recovered. Nuclei from four samples were mixed resulting in a total of six pools. Each pool containing 24,000 nuclei were loaded on a 10X reaction using 10X Genomics Chromium single-cell 3′ reagent kits (version 3.1). Single-cell cDNA libraries were produced according to the manufacturer’s instructions and sequenced on an Illumina NovaSeq 6000 (Illumina, San Diego, CA) using 2x S1 flow cells to obtain 2,130,447,680 reads for pool 1 and 2,135,194,048 reads for pool 2. After all filtration steps and quality controls, there were approximately 70 million reads per sample.

#### Data analysis

BCL files were converted to Fastq using bcl2fastq v. 2.19.0 (Illumina). Salmon Alevin v1.9.0 ^11^ and Alevin-fry v0.7.0 ^12^ were used to perform pseudo-alignment of the RNA and hashtag oligos (HTO) library. The RNA library was pseudo-aligned to a custom reference transcriptome, distinguishing between spliced and unspliced transcripts, modified from the ensemble GRCm38 reference, using the salmon index tool (version 1.5.0) ^11^. Following pseudo-alignment, mapping steps were performed using the Alevin-fry framework (v.0.7.0) ^13^ and cells were loaded into R. Barcode ranks were determined from the unfiltered, unspliced counts using the barcodeRanks function from the DropletUtils package (v1.10.3), and ranks were used to determine knee point and inflection point. Subsequently, a Hartigan’s Dip test was performed on counts above the knee point to assess if data were unimodal. If data were found to be unimodal, they were further analyzed for cells above the calculated inflection point. If data were not unimodal, a bimodal model was fitted to the counts from cells above the inflection with the mclust package (v.5.4.7) ^14^. Barcode ranks were calculated, and further analysis was performed on cells above the re-calculated inflection. Ensemble gene IDs were mapped to symbol using the AnnotationDbi package (v.1.52.0). Cells overlapping between RNA and HTO libraries were imported into Seurat version 4.1.1 ^15^. The HTO read count assay was subjected to centered log-ratio normalization to account for variation in sequencing depth. Normalized HTO read counts were modeled for each HTO using the mclust R package ^14^, and assigned a HTO label based on the presence of a higher number of normalized HTO read counts. Cells were grouped into sample identities based on the number of HTO assignments as either negatives, singlets and doublets, and tSNE was run to visualize proportions and clustering of the three groups. Seurat objects were subsequently converted to a singleCellExperiment, v(1.12.0) ^16^. RNA counts were log-normalized and subjected to principal component analysis (PCA). Intra-sample doublets were inferred from inter-sample doublets with the scDblFinder package (v.1.4.0). All lane results were aggregated into a single object, genes without detection were removed, and negatives, doublets, and inferred doublets were removed. The object was subsequently normalized using SCTransform ^17^ and subjected to PCA. Global dimensions were calculated using the intrinsicDimension R package (v.1.2.0). The object was subsequently subjected to Uniform Manifold Approximation and Projection (UMAP), k.param nearest neighbor’s computation, and cluster identification using Seurat. Marker genes were identified using the FindMarkers function from Seurat. Cell populations were identified based on marker enrichment by cross-referencing the murine liver cell atlas (livercellatlas.org, ^18^). For analysis of sub-groups, groups were subset and subjected to principal component analysis followed by uniform manifold approximation and projection reduction, neighbor calculation, and clustering for 35 principal components. Markers were identified as described above.

## References

1. Dall M, Hassing AS, Niu L, et al. Hepatocyte-specific perturbation of NAD+ biosynthetic pathways in mice induces reversible nonalcoholic steatohepatitis–like phenotypes. *The Journal of Biological Chemistry*. 2021;297(6):101388-101388. doi:10.1016/J.JBC.2021.101388

2. Dall M, Trammell SAJ, Asping M, et al. Mitochondrial function in liver cells is resistant to perturbations in NAD + salvage capacity. *Journal of Biological Chemistry*. 2019:jbc.RA118.006756-jbc.RA118.006756. doi:10.1074/jbc.ra118.006756

3. Rappsilber J, Ishihama Y, Mann M. Stop and go extraction tips for matrix-assisted laser desorption/ionization, nanoelectrospray, and LC/MS sample pretreatment in proteomics. *Analytical chemistry*. 2003;75(3):663-670. doi:10.1021/AC026117I

4. Bache N, Geyer PE, Bekker-Jensen DB, et al. A Novel LC System Embeds Analytes in Pre-formed Gradients for Rapid, Ultra-robust Proteomics. *Molecular & cellular proteomics : MCP*. 2018;17(11):2284-2296. doi:10.1074/MCP.TIR118.000853

5. Meier F, Brunner AD, Frank M, et al. diaPASEF: parallel accumulation-serial fragmentation combined with data-independent acquisition. *Nature methods*. 2020;17(12):1229-1236. doi:10.1038/S41592-020-00998-0

6. Meier F, Brunner AD, Koch S, et al. Online Parallel Accumulation-Serial Fragmentation (PASEF) with a Novel Trapped Ion Mobility Mass Spectrometer. *Molecular & cellular proteomics : MCP*. 2018;17(12):2534-2545. doi:10.1074/MCP.TIR118.000900

7. Demichev V, Szyrwiel L, Yu F, et al. dia-PASEF data analysis using FragPipe and DIA-NN for deep proteomics of low sample amounts. *Nature communications*. 2022;13(1)doi:10.1038/S41467-022-31492-0

8. Ritchie ME, Phipson B, Wu DI, et al. Limma powers differential expression analyses for RNA-sequencing and microarray studies. *Nucleic Acids Research*. 2015;43(7):e47-e47. doi:10.1093/nar/gkv007

9. Yu G, Wang LG, Han Y, He QY. ClusterProfiler: An R package for comparing biological themes among gene clusters. *OMICS A Journal of Integrative Biology*. 2012;16(5):284-287. doi:10.1089/omi.2011.0118

10. Stoeckius M, Zheng S, Houck-Loomis B, et al. Cell Hashing with barcoded antibodies enables multiplexing and doublet detection for single cell genomics. *Genome Biology*. 2018;19(1):224-224. doi:10.1186/s13059-018-1603-1

11. Patro R, Duggal G, Love MI, Irizarry RA, Kingsford C. Salmon provides fast and bias-aware quantification of transcript expression. *Nature methods*. 2017;14(4):417-419. doi:10.1038/NMETH.4197

12. Srivastava A, Malik L, Smith T, Sudbery I, Patro R. Alevin efficiently estimates accurate gene abundances from dscRNA-seq data. *Genome Biology*. 2019;20(1):65-65. doi:10.1186/s13059-019-1670-y

13. He D, Zakeri M, Sarkar H, Soneson C, Srivastava A, Patro R. Alevin-fry unlocks rapid, accurate and memory-frugal quantification of single-cell RNA-seq data. *Nature methods*. 2022;19(3):316-322. doi:10.1038/S41592-022-01408-3

14. Scrucca L, Fop M, Murphy TB, Raftery AE. mclust 5: Clustering, Classification and Density Estimation Using Gaussian Finite Mixture Models. *The R journal*. 2016;8(1):289-289. doi:10.32614/rj-2016-021

15. Hao Y, Hao S, Andersen-Nissen E, et al. Integrated analysis of multimodal single-cell data. *Cell*. 2021;184(13):3573-3587.e29. doi:10.1016/J.CELL.2021.04.048

16. Amezquita RA, Lun ATL, Becht E, et al. Orchestrating single-cell analysis with Bioconductor. *Nature methods*. 2020;17(2):137-145. doi:10.1038/S41592-019-0654-X

17. Hafemeister C, Satija R. Normalization and variance stabilization of single-cell RNA-seq data using regularized negative binomial regression. *Genome Biology*. 2019;20(1)doi:10.1186/S13059-019-1874-1

18. Guilliams M, Bonnardel J, Haest B, et al. Spatial proteogenomics reveals distinct and evolutionarily conserved hepatic macrophage niches. *Cell*. 2022;185(2):379-396.e38. doi:10.1016/j.cell.2021.12.018
