# Supplementary material for: Hepatocyte dedifferentiation in 2D culture reveals extensive transcriptomic and proteomic rewiring
Source: Hepatol Commun. 2025 Oct 7;9(11):e0795. doi: 10.1097/HC9.0000000000000795 (PMC12506984; doi:10.1097/HC9.0000000000000795)
Supplement: Supplementary file 3 [file hc9-9-e0795-s003.pdf]

Supporting Fig. 1

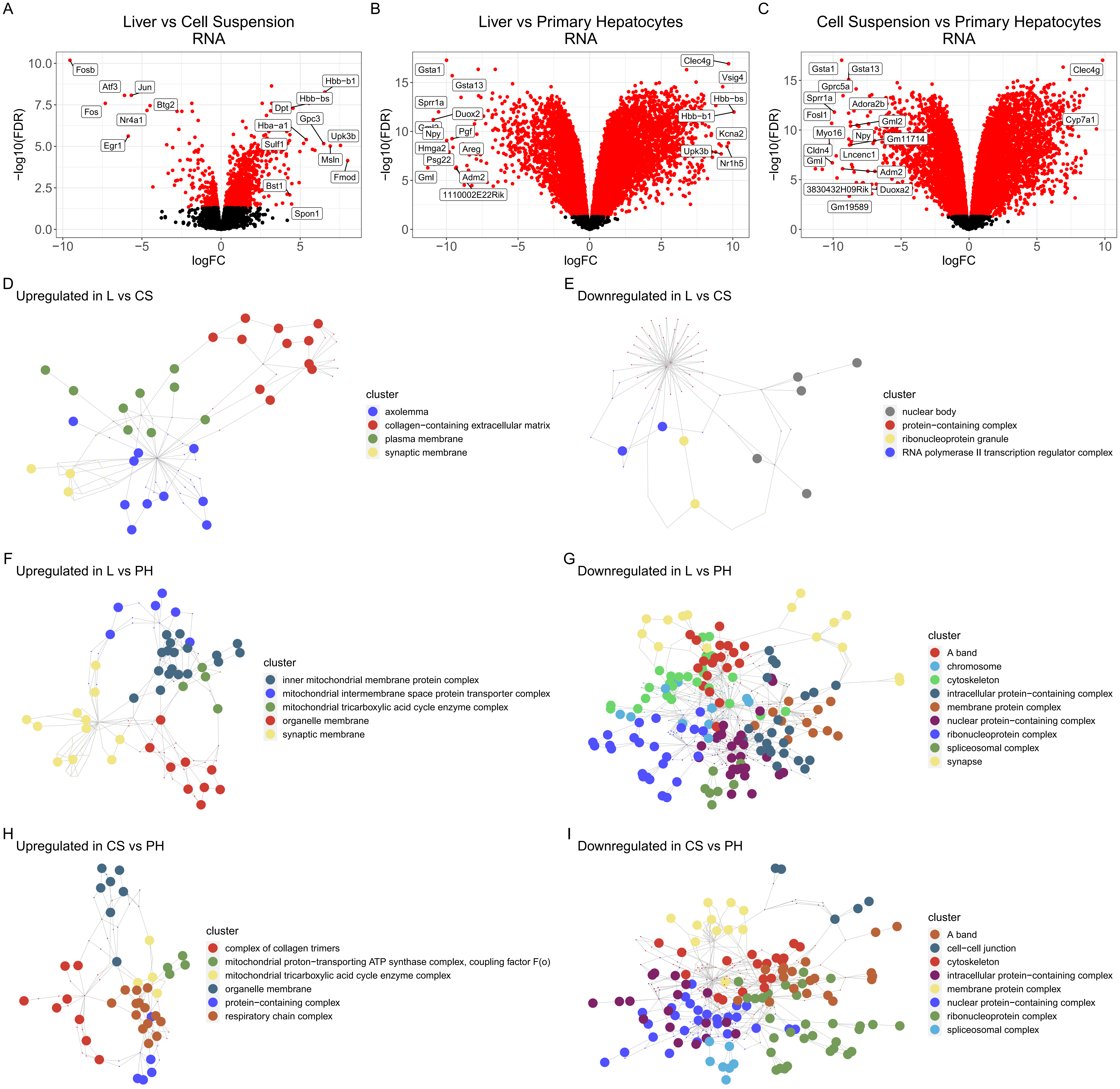

Figure S1: Culturing decreases expression of mitochondrial genes in primary hepatocytes

A-C: Volcano plots of bulk mRNA-seq data, comparing –log10(FDR) and log fold change values. The 20 genes with the largest absolute fold change and a symbol annotation are labeled for each plot. Points were colored red when FDR<0.05. D-I: Network graphs showing clustering results of significantly enriched GO terms from the cellular component ontology. Large dots indicate significant enrichment. GO terms were clustered using the Leiden algorithm at resolution 0.6.
