# Supplementary figures and images for: Hepatocyte dedifferentiation in 2D culture reveals extensive transcriptomic and proteomic rewiring
Source: Hepatol Commun. 2025 Oct 7;9(11):e0795. doi: 10.1097/HC9.0000000000000795 (PMC12506984; doi:10.1097/HC9.0000000000000795)

Supporting Fig. 2

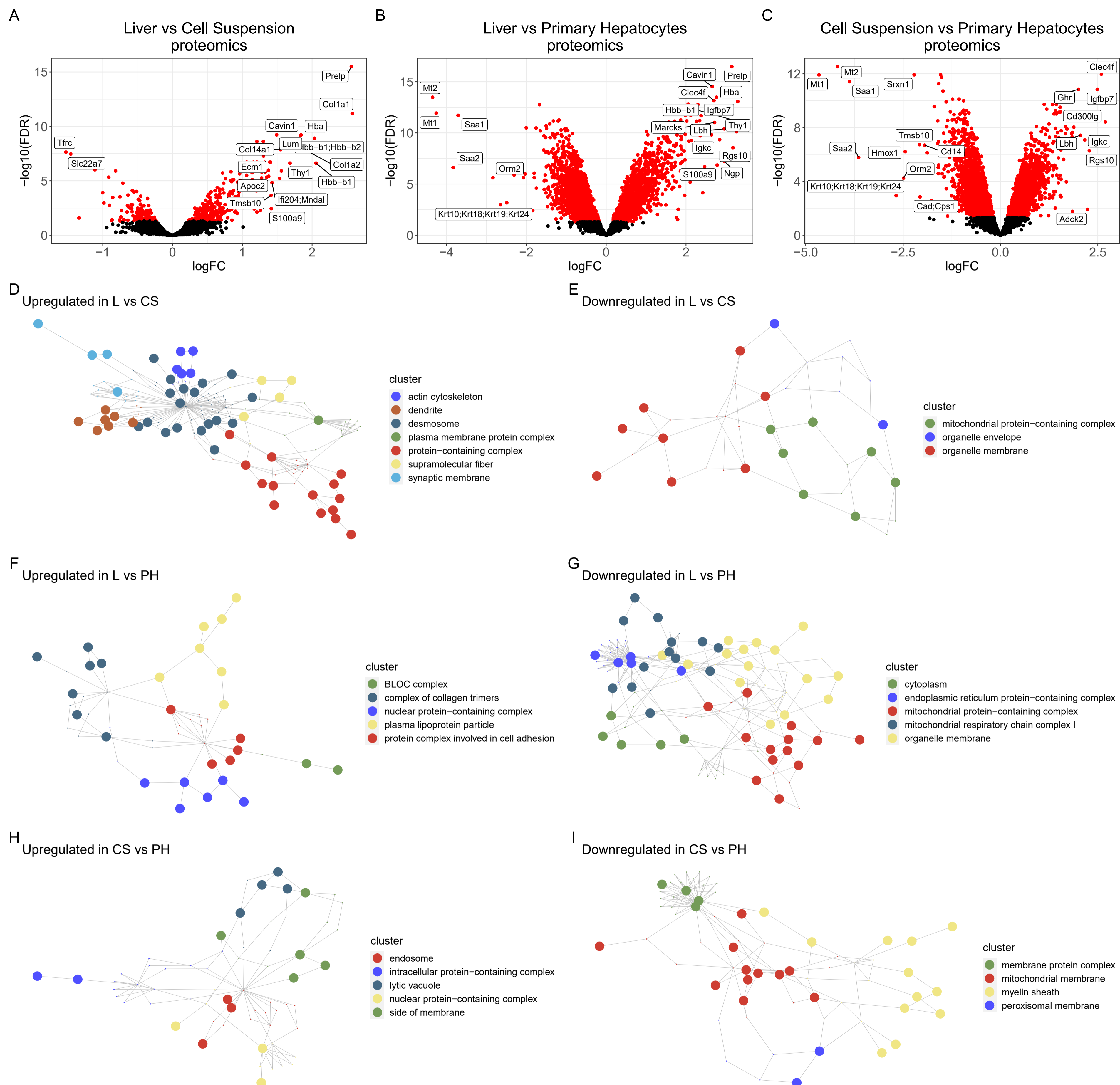

Supplement: Supplementary file 6 [file hc9-9-e0795-s006.pdf]
