# Supplementary material for: Hepatocyte dedifferentiation in 2D culture reveals extensive transcriptomic and proteomic rewiring
Source: Hepatol Commun. 2025 Oct 7;9(11):e0795. doi: 10.1097/HC9.0000000000000795 (PMC12506984; doi:10.1097/HC9.0000000000000795)
Supplement: Supplementary file 8 [file hc9-9-e0795-s008.pdf]

Supporting Fig. 3

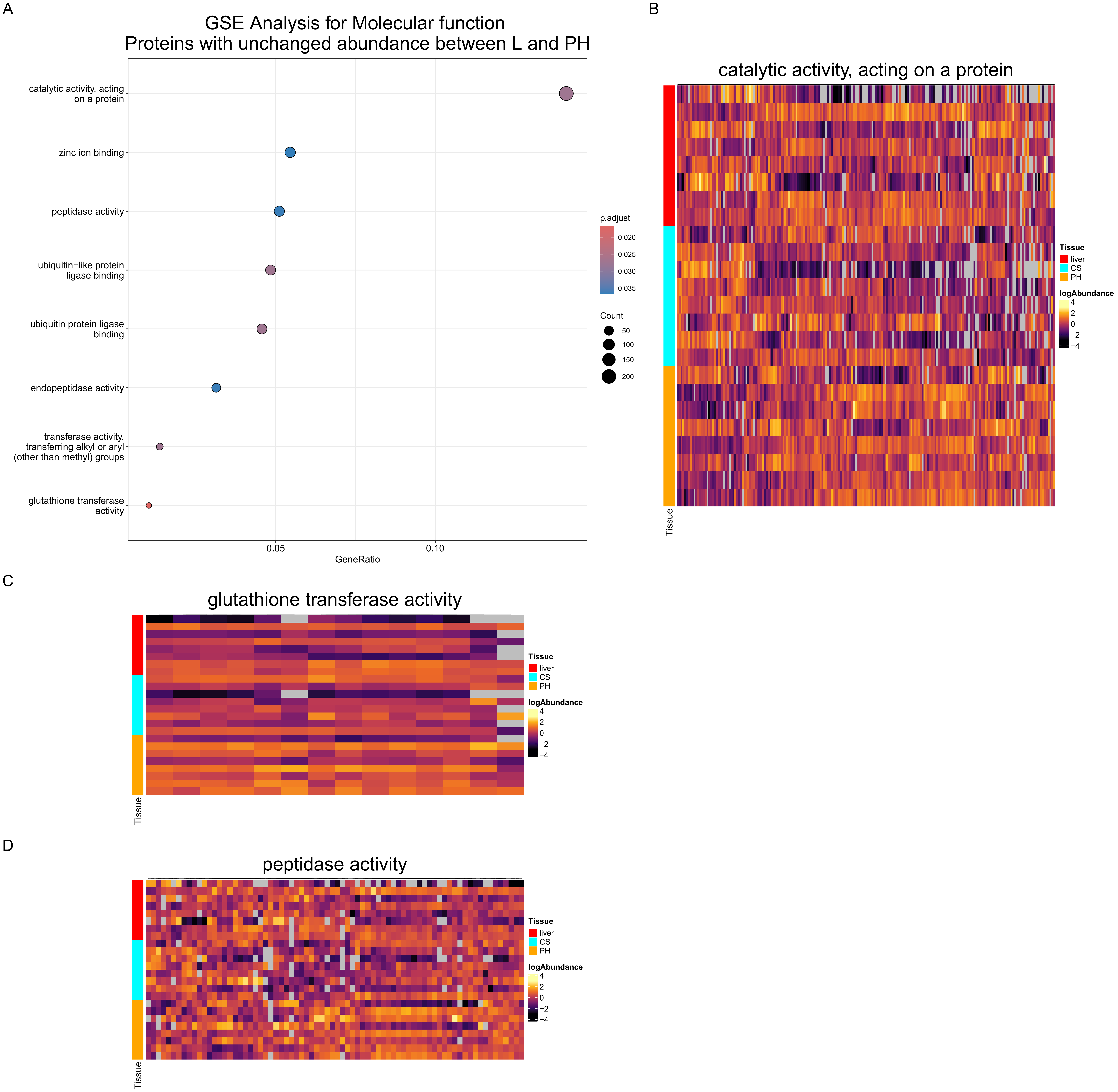

Figure S3: Cultured hepatocytes maintain abundance of proteins enriched for peptidase activity
A. Dotplot showing the result of gene ontology enrichment analysis on the Molecular Function ontology for proteins with unaltered abundance between L and PH. B. Heatmap of proteins with an unaltered abundance from the “catalytic activity, acting on a protein” ontology. C. Heatmap of proteins with an unaltered abundance from the “glutathione transferase activity” ontology. D. Heatmap of proteins with an unaltered abundance from the “peptidase activity” ontology.
