# Supplementary material for: Hepatocyte dedifferentiation in 2D culture reveals extensive transcriptomic and proteomic rewiring
Source: Hepatol Commun. 2025 Oct 7;9(11):e0795. doi: 10.1097/HC9.0000000000000795 (PMC12506984; doi:10.1097/HC9.0000000000000795)
Supplement: Supplementary file 12 [file hc9-9-e0795-s012.pdf]

Supporting Fig. 4

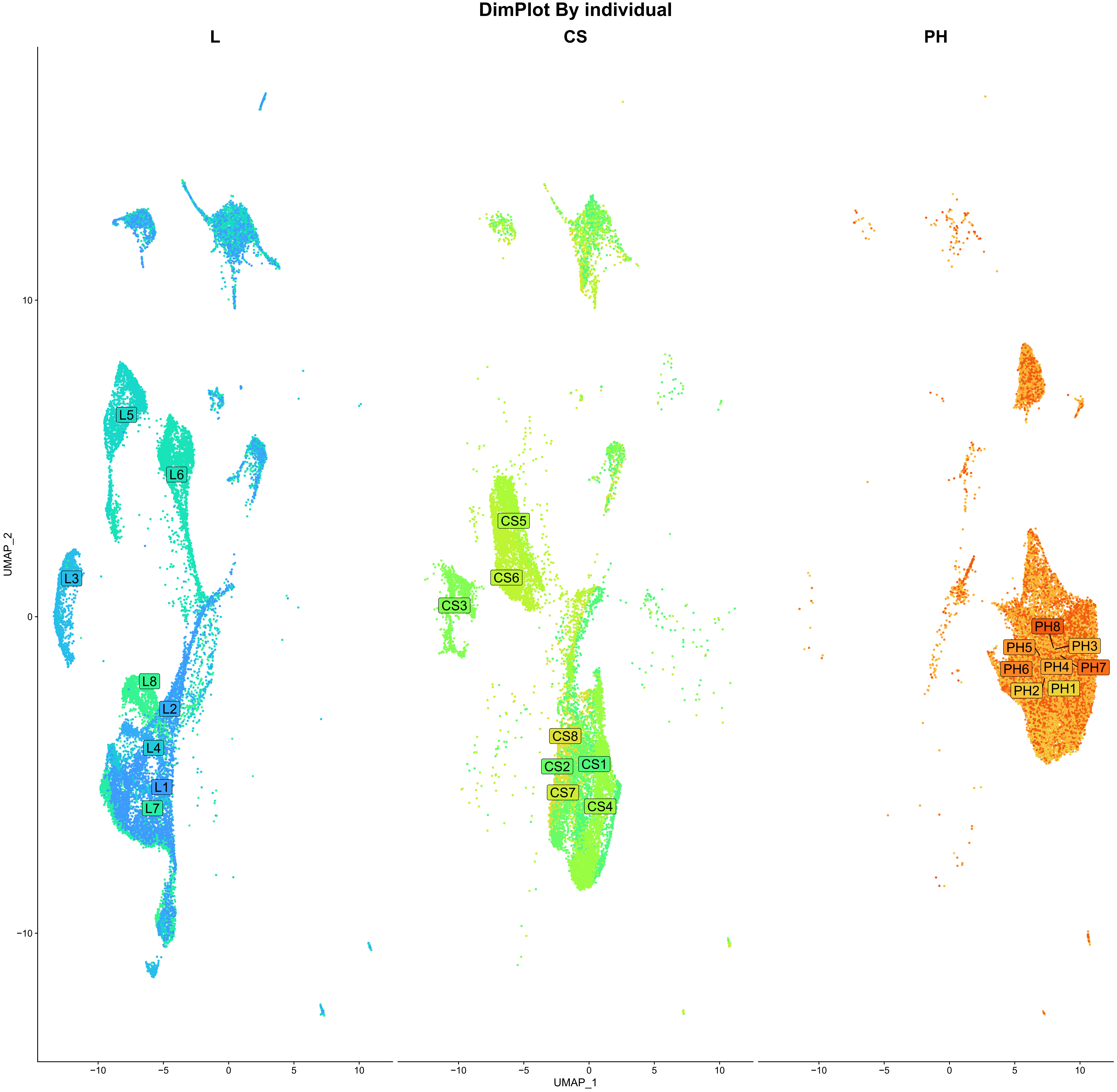

Figure S4: Single nucleus RNAseq analysis confirms decreased homogeneity in cultured hepatocytes vs. freshly-isolated cells
UMAP plot split by sample type, colored by Sample ID for dimensions 1 and 2.
