# Supplementary material for: Hepatocyte dedifferentiation in 2D culture reveals extensive transcriptomic and proteomic rewiring
Source: Hepatol Commun. 2025 Oct 7;9(11):e0795. doi: 10.1097/HC9.0000000000000795 (PMC12506984; doi:10.1097/HC9.0000000000000795)
Supplement: Supplementary file 13 [file hc9-9-e0795-s013.pdf]

Supporting Fig. 5

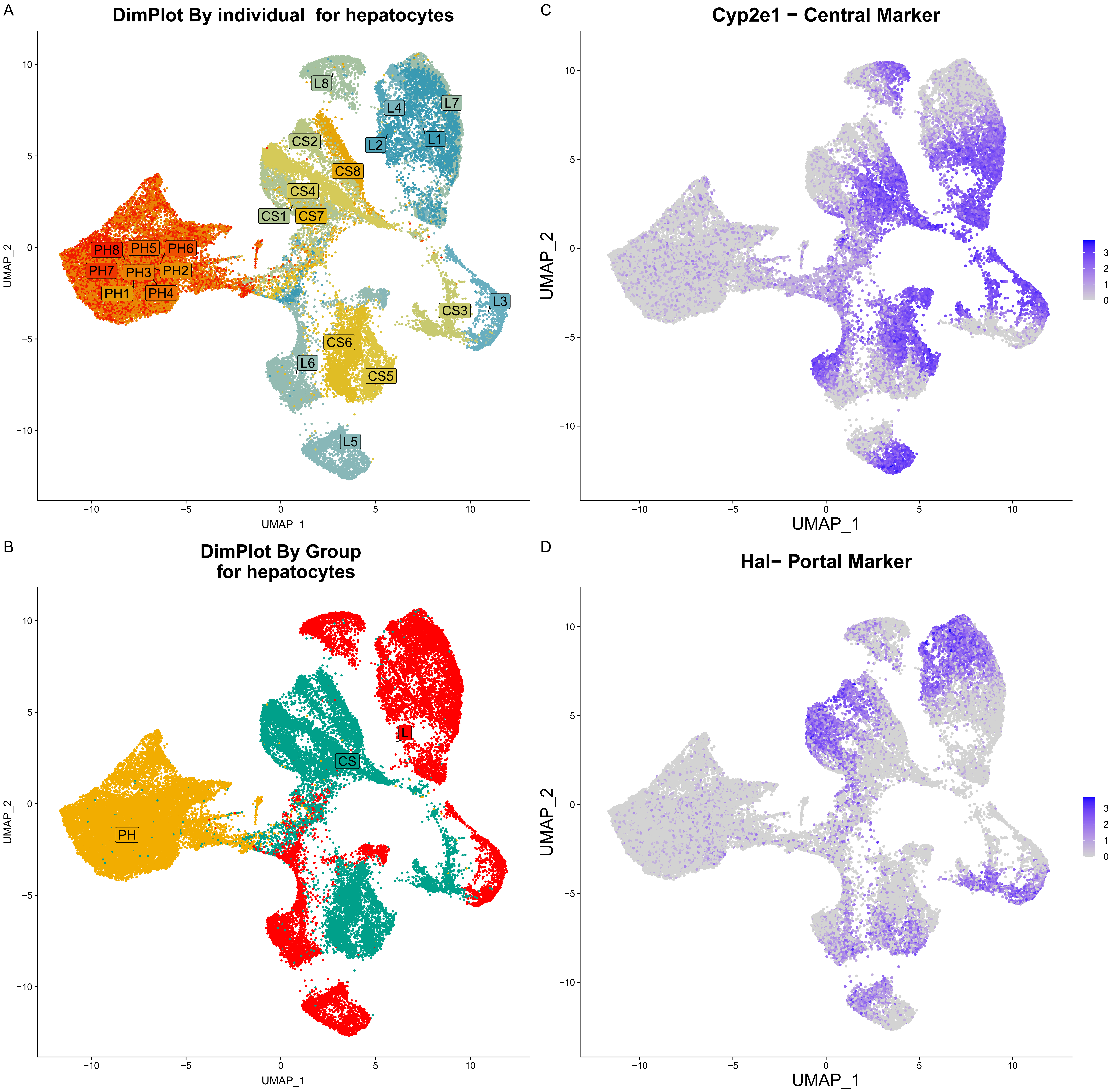

Figure S5: Single nucleus RNAseq analysis of hepatocyte sub-populations confirms increased homogeneity in hepatocytes following 24h of culturing

A. UMAP plot for dimension 1 and 2, showing clustering of hepatocyte populations, colored by sample ID. B. UMAP plot for dimension 1 and 2 for hepatocyte populations colored by group. C. Feature plot showing expression of the central vein-associated marker gene cytochrome p450 2e1 (Cyp2e1). Feature plot showing expression of the portal-vein associated marker gene histidine ammonia lyase (Hal). L: Liver. CS: Cell suspension. PH: Primary hepatocytes cultured for 24h.
