# Supplementary material for: Hepatocyte dedifferentiation in 2D culture reveals extensive transcriptomic and proteomic rewiring
Source: Hepatol Commun. 2025 Oct 7;9(11):e0795. doi: 10.1097/HC9.0000000000000795 (PMC12506984; doi:10.1097/HC9.0000000000000795)
Supplement: Supplementary file 16 [file hc9-9-e0795-s016.pdf]

Supporting Figure 6

A

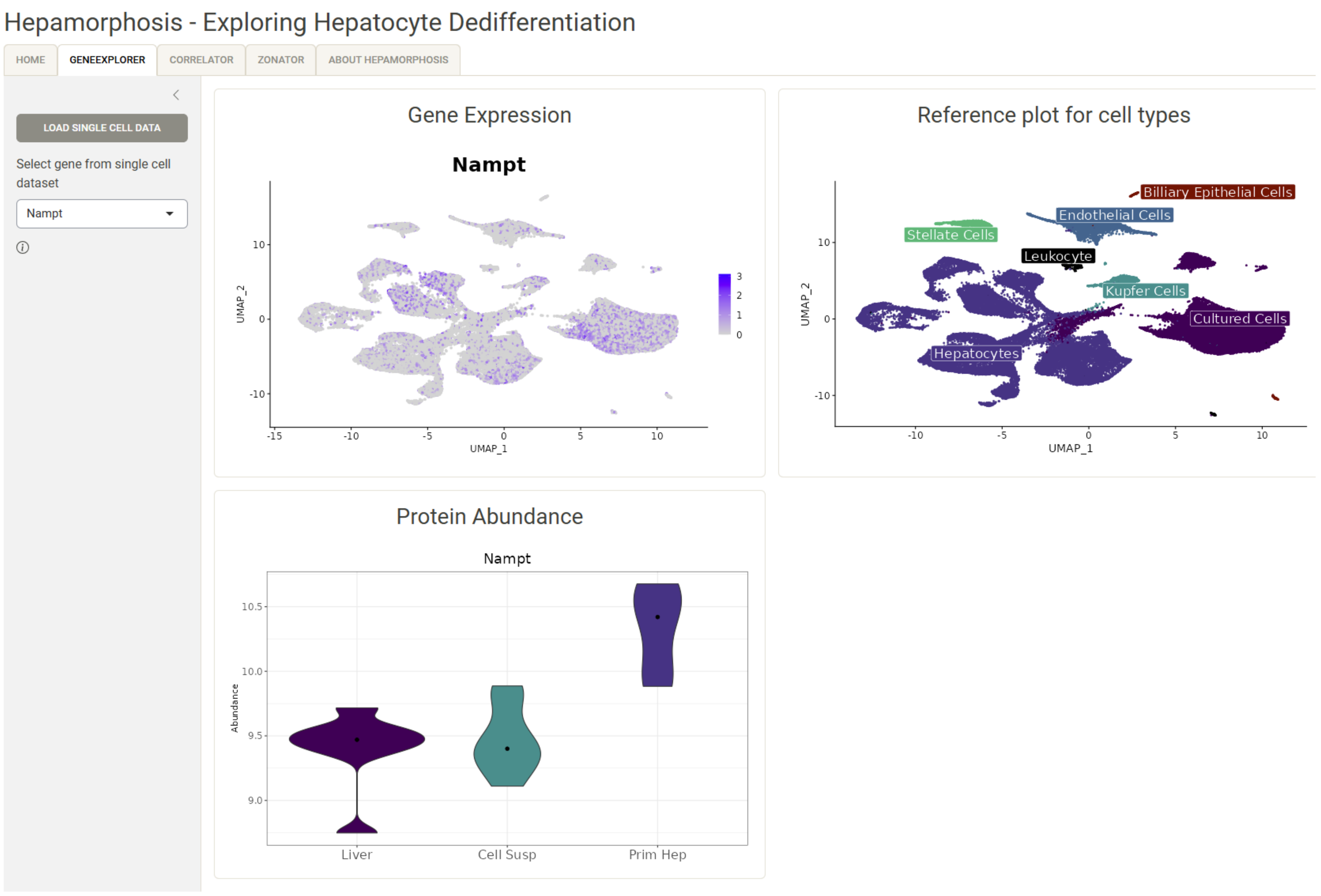

B

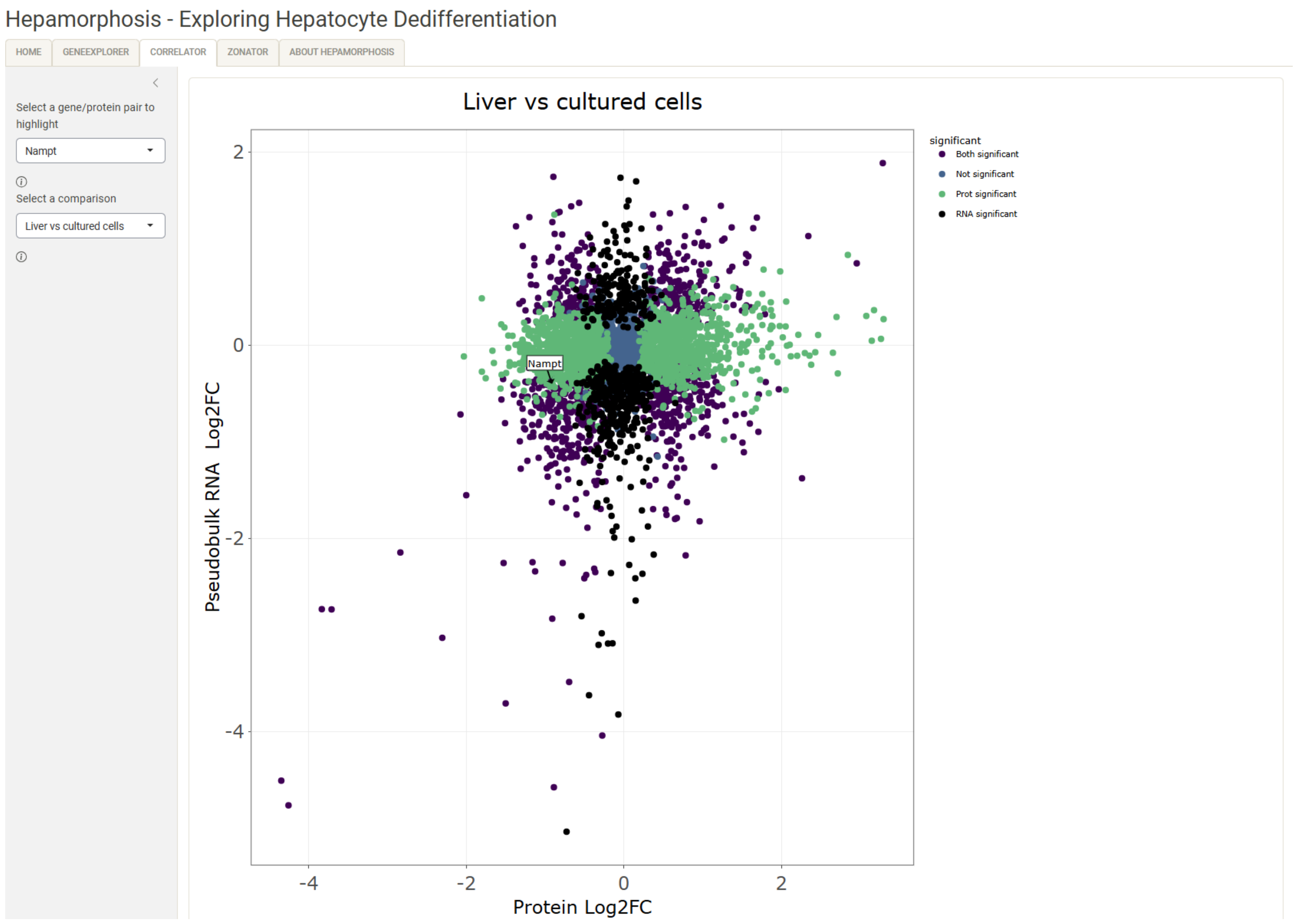

C

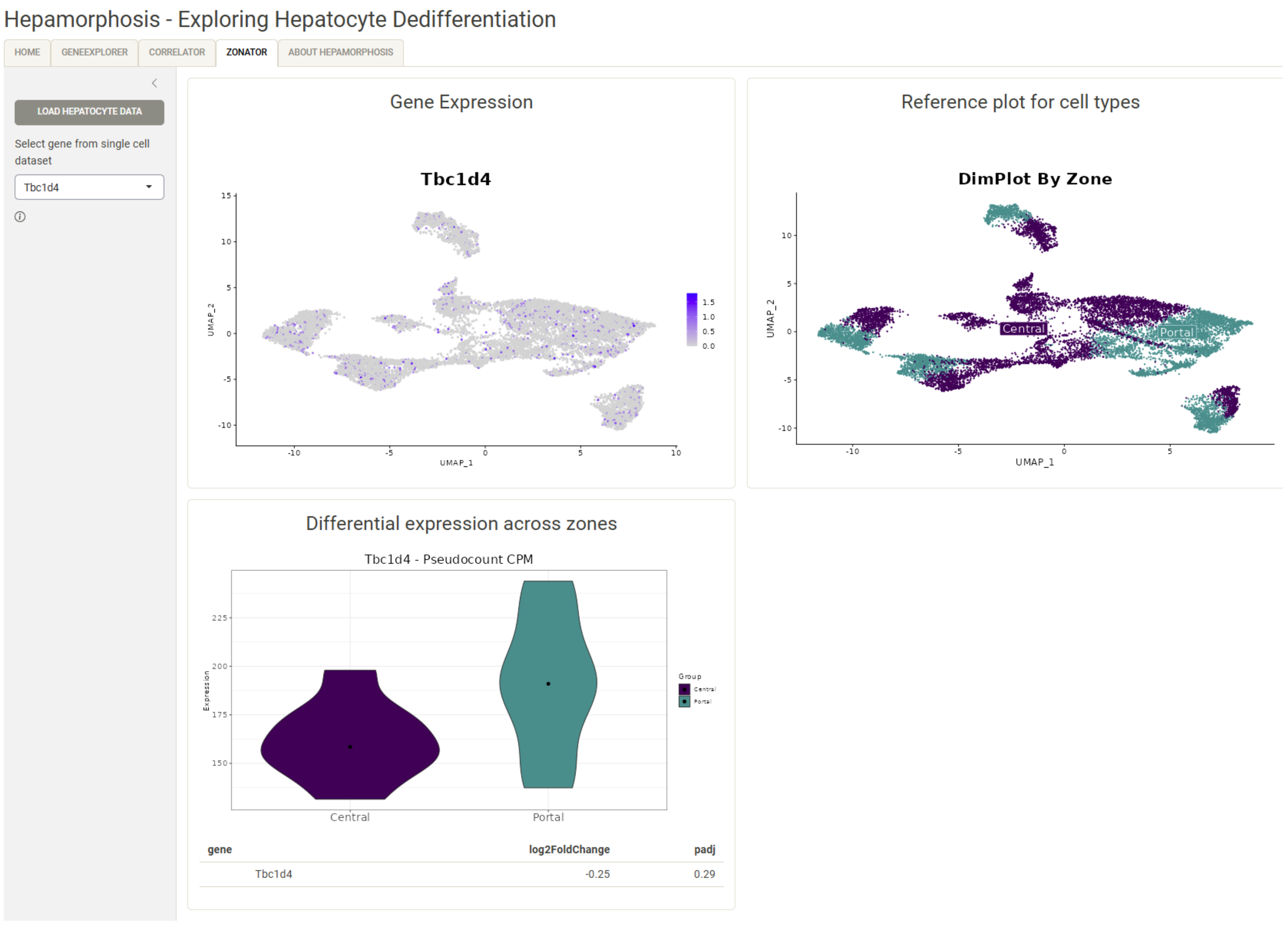

Figure S6: The app "Hepamorphosis" allows users to explore hepatocyte dedifferentiation on their own
A. "Geneexplorer" allows the user to select a gene found in the snRNAseq dataset. Gene expression will be displayed as a Feature Plot and if the target is found in the proteomics dataset, abundance in Liver, Cell Suspension and Primary Hepatocytes will be displayed as a violin plot. B. "Correlator" allows the user to search target genes/proteins found in both the snRNAseq- and the proteomics dataset and see how well expression and abundance correlates. C. "Zonator" allows the user to search genes found in the snRNAset dataset and assess the potential zonation of the target.
