# Supplementary material for: Hepatocyte dedifferentiation in 2D culture reveals extensive transcriptomic and proteomic rewiring
Source: Hepatol Commun. 2025 Oct 7;9(11):e0795. doi: 10.1097/HC9.0000000000000795 (PMC12506984; doi:10.1097/HC9.0000000000000795)
Supplement: Supplementary file 17 [file hc9-9-e0795-s017.pdf]

Supporting Figure 7

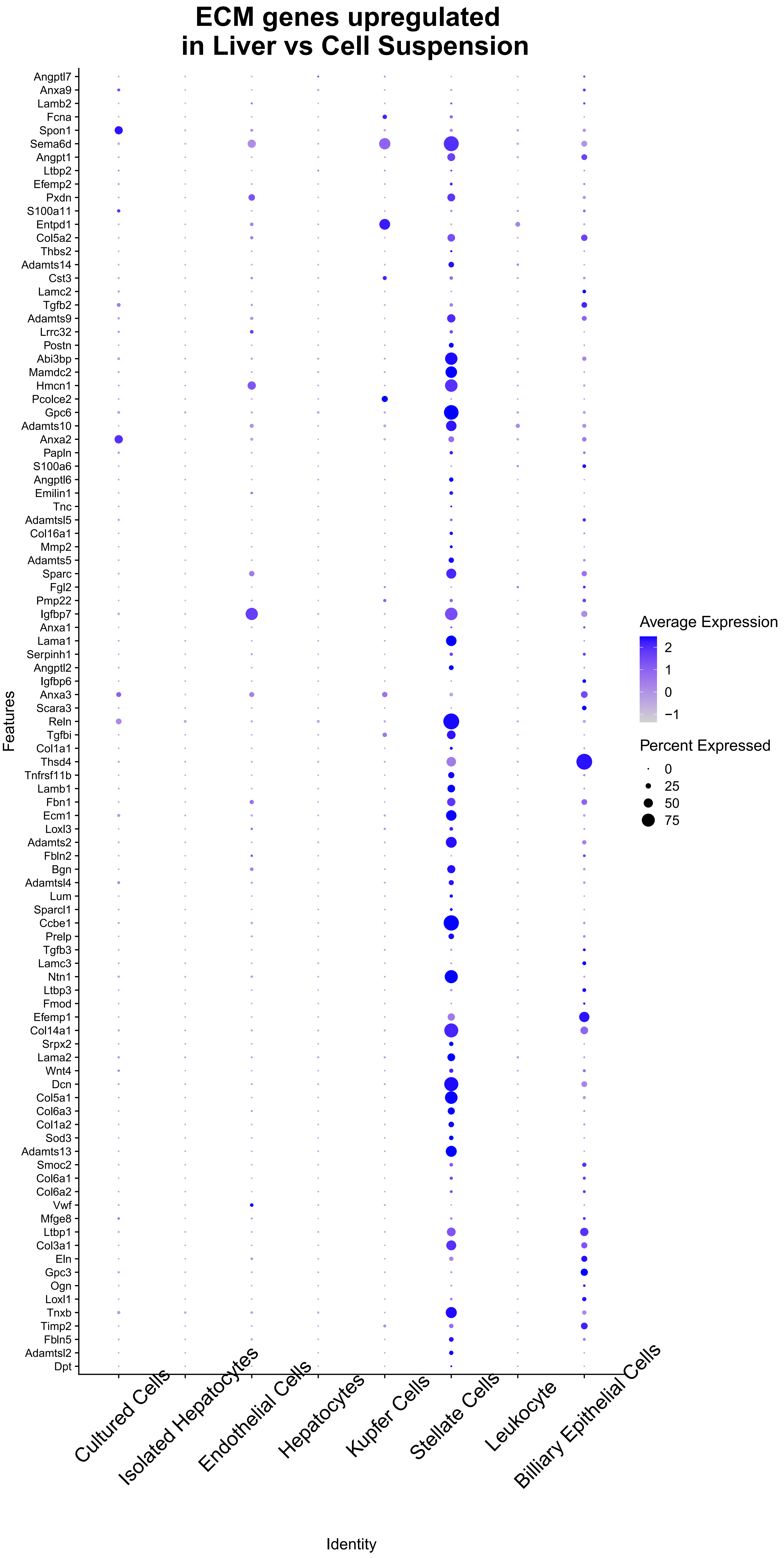

Figure S7: Genes from the “extracellular matrix” ontology are highly expressed in the stellate cell cluster
Dotplot of differentially expressed genes for liver vs. cell suspension belonging to the “Extracellular Matrix” ontology split by cell type
